# Single-cell and spatial transcriptomics identify a macrophage population associated with skeletal muscle fibrosis

**DOI:** 10.1126/sciadv.add9984

**Published:** 2023-07-07

**Authors:** Gerald Coulis, Diego Jaime, Christian Guerrero-Juarez, Jenna M. Kastenschmidt, Philip K. Farahat, Quy Nguyen, Nicholas Pervolarakis, Katherine McLinden, Lauren Thurlow, Saba Movahedi, Brandon S. Hughes, Jorge Duarte, Andrew Sorn, Elizabeth Montoya, Izza Mozaffar, Morgan Dragan, Shivashankar Othy, Trupti Joshi, Chetan P. Hans, Virginia Kimonis, Adam L. MacLean, Qing Nie, Lindsay M. Wallace, Scott Q. Harper, Tahseen Mozaffar, Marshall W. Hogarth, Surajit Bhattacharya, Jyoti K. Jaiswal, David R. Golann, Qi Su, Kai Kessenbrock, Michael Stec, Melissa J. Spencer, Jesse R. Zamudio, S. Armando Villalta

**Affiliations:** ^1^ Department of Physiology and Biophysics, University of California Irvine, Irvine, CA, USA.; ^2^ Institute for Immunology, University of California Irvine, Irvine, CA, USA.; ^3^ Carle Illinois College of Medicine, University of Illinois at Urbana-Champaign, Champaign, IL, USA.; ^4^ Department of Biological Chemistry, University of California Irvine, Irvine, CA USA.; ^5^ Department of Molecular Cell and Developmental Biology, University of California Los Angeles, Los Angeles, CA, USA.; ^6^ Department of Health Management and Informatics, University of Missouri, Columbia, MO, USA.; ^7^ Department of Cardiovascular Medicine, University of Missouri, Columbia, MO USA.; ^8^ Department of Pediatrics, University of California Irvine, Irvine, CA, USA.; ^9^ Department of Quantitative and Computational Biology, University of Southern California, Los Angeles, CA, USA.; ^10^ Department of Mathematics, Department of Developmental and Cell Biology, University of California Irvine, Irvine, CA, USA.; ^11^ Center for Gene Therapy, The Abigail Wexner Research Institute at Nationwide Children’s Hospital, Columbus, OH, USA.; ^12^ Department of Pediatrics, The Ohio State University, Columbus, OH, USA.; ^13^ Department of Neurology, University of California Irvine, Irvine, CA, USA.; ^14^ Department of Pathology and Laboratory Medicine, University of California Irvine, Irvine, CA, USA.; ^15^ Children’s National Hospital, Research Center for Genetic Medicine, Washington, DC, USA.; ^16^ Regeneron Pharmaceuticals Inc., Tarrytown, NY, USA.; ^17^ Department of Neurology, University of California Los Angeles, Los Angeles, CA, USA.

## Abstract

Macrophages are essential for skeletal muscle homeostasis, but how their dysregulation contributes to the development of fibrosis in muscle disease remains unclear. Here, we used single-cell transcriptomics to determine the molecular attributes of dystrophic and healthy muscle macrophages. We identified six clusters and unexpectedly found that none corresponded to traditional definitions of M1 or M2 macrophages. Rather, the predominant macrophage signature in dystrophic muscle was characterized by high expression of fibrotic factors, galectin-3 (gal-3) and osteopontin (*Spp1*). Spatial transcriptomics, computational inferences of intercellular communication, and in vitro assays indicated that macrophage-derived Spp1 regulates stromal progenitor differentiation. Gal-3^+^ macrophages were chronically activated in dystrophic muscle, and adoptive transfer assays showed that the gal-3^+^ phenotype was the dominant molecular program induced within the dystrophic milieu. Gal-3^+^ macrophages were also elevated in multiple human myopathies. These studies advance our understanding of macrophages in muscular dystrophy by defining their transcriptional programs and reveal *Spp1* as a major regulator of macrophage and stromal progenitor interactions.

## Teaser

Transcriptomics identifies a Spp1-mediated muscle macrophage and stromal progenitor interaction associated with muscle fibrosis.

## INTRODUCTION

Macrophages have a central role in innate immunity and contribute to tissue homeostasis by regulating tissue repair and remodeling of the extracellular matrix (ECM) (*1*). Their diverse functions within the tissue are matched by a high degree of molecular heterogeneity, reflecting microenvironmental adaptability (*2*). Skeletal muscle macrophages comprise resident and monocyte-derived populations, the latter infiltrating upon muscle injury or disease (*3*). The M1 and M2 macrophage paradigm (*4*) has been used to describe the functional heterogeneity of skeletal muscle macrophages (*5*). In acute muscle trauma, proinflammatory M1 macrophages initially infiltrate injured muscle to phagocytose cellular debris and activate muscle stem cells (*6*). The subsequent transition to M2 macrophages in the regenerative phase promotes muscle stem cell differentiation and the resolution of inflammation (*3*, *7*). M1- and M2-like macrophages have been described in Duchenne muscular dystrophy (DMD) (*7*). However, growing evidence indicates that the regulation and functional role of macrophages are more complex in the context of chronic muscle degenerative diseases such as DMD.

Although macrophages are essential for muscle repair following acute trauma, their dysregulation promotes the pathogenesis of DMD, a lethal form of muscular dystrophy caused by mutations in the *DMD* gene (*8*). The transition from M1 to M2 macrophages seen in acute injury is disrupted in the X-linked muscular dystrophy (mdx) mouse model of DMD by asynchronous bouts of muscle injury and regeneration (*9*). Consequently, M1 macrophages are chronically activated and promote muscle injury in an inducible nitric oxide synthase–dependent manner (*10*), and the reparative function of M2 macrophages is pathologically repurposed to promote fibrosis. This impairment is expected to induce macrophage transcriptional programs that are distinct from those induced in acute injury and contribute to muscle pathology. In support of this, several studies reported that perturbing macrophage function and/or activation impaired regeneration and promoted fibrosis in muscular dystrophy (*11*–*13*), Pompe disease (*14*), and dysferlinopathy (*15*).

Fibrosis is the aberrant accumulation of collagen and other ECM proteins in chronically inflamed tissues, leading to organ failure and death (*16*). Fibro/adipogenic progenitors (FAPs) are stromal cells that give rise to fibroblasts and adipocytes and regulate muscle repair and fibrosis (*17*). Following acute injury, FAPs expand and contribute to muscle repair by facilitating myogenesis and ECM formation (*18*). Fibrosis is mitigated by infiltrating macrophages that clear FAPs through tumor necrosis factor–α–mediated apoptosis (*19*). Conversely, transforming growth factor–β (TGF-β), which is highly up-regulated in dystrophic muscle (*20*), inhibits FAP apoptosis and guides their differentiation into matrix-producing myofibroblasts (*17*). In this setting, persistence of FAPs and their skewed differentiation toward fibroblasts contributes to the development of muscle fibrosis. Unexpectedly, inhibition of TGF-β does not fully restore FAP clearance (*19*), indicating that there are additional factors that promote FAP differentiation and/or survival. Osteopontin (*Spp1*) is a potential candidate because it is highly expressed by macrophages (*21*), elevated in dystrophic muscle in patients with DMD (*22*), and promotes fibrosis in muscular dystrophy (*22*, *23*). However, the repertoire of profibrotic factors, including *Spp1*, expressed by dystrophic muscle macrophages and their cellular targets has not been fully defined.

In this study, we used an unbiased single-cell RNA sequencing (scRNAseq) approach to define the transcriptional profiles of macrophages from normal and dystrophic muscle. The scRNAseq identified several macrophage populations with transcriptomes not previously associated with muscular dystrophy. We focused on three populations that corresponded to resident macrophages, monocyte-derived macrophages (MDMs), and a population characterized by high expression of profibrotic factors, *Lgals3* [galectin-3 (gal-3)] (*24*) and *Spp1* (*25*). Given the selective induction of *Lgals3* and *Spp1* in dystrophic muscle macrophages, we hypothesize that this transcriptional profile defines a fibrogenic macrophage that promotes fibrosis during muscular dystrophy.

We demonstrate that gal-3^+^ macrophages are activated in response to acute injury and are elevated in several muscle disorders. Although this macrophage population is transient following acute injury, gal-3^+^ macrophages are chronically activated during muscular dystrophy. Spatial transcriptomic analysis of dystrophic muscle revealed that areas enriched in gal-3^+^ macrophages and stromal cells expressed genes associated with muscle fibrosis. Furthermore, gal-3^+^ macrophages colocalize with stromal cells in dystrophic lesions, and computational analysis with CellChat shows that *Spp1* mediates communication between these cell types. Collectively, results from this study identify a distinct transcriptional profile in dystrophic muscle macrophages that is associated with fibrosis, revealing the potential for therapeutic strategies that target fibrogenic macrophages in muscular dystrophy.

## RESULTS

### scRNAseq reveals unique cell states within skeletal muscle macrophages

**Fig. 1.**
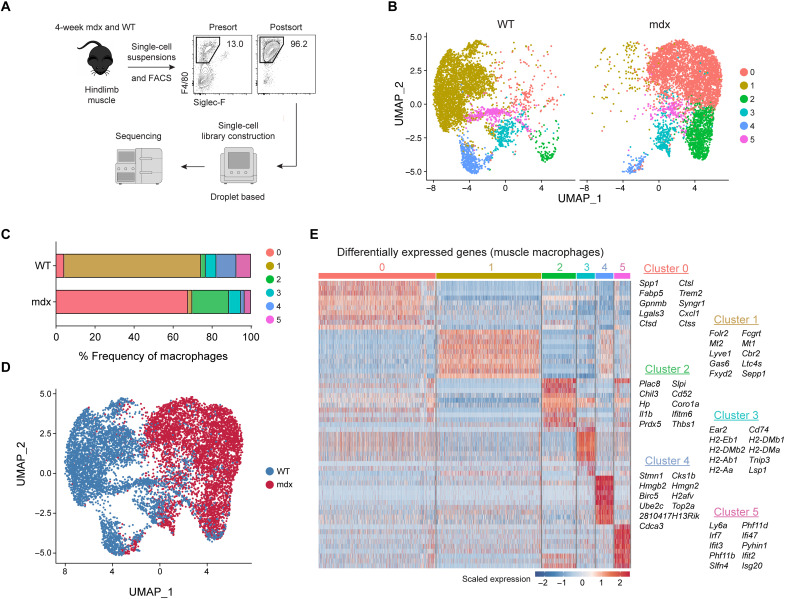
Identification of transcriptomic diversity in skeletal muscle macrophages by scRNAseq. (**A**) Muscle macrophages were isolated from 4-week-old WT (healthy) and B10.mdx (dystrophic) mice and analyzed by scRNAseq. *n* = 1 (**B**) Dimensionality reduction via UMAP of healthy or dystrophic muscle macrophages. (**C**) Proportion of WT or mdx muscle macrophage clusters. (**D**) Identities classified by genotype. (**E**) Differential gene expression analysis showing the top 10 most differentially expressed genes (DEGs) for each cluster.

scRNAseq was performed to unbiasedly phenotype skeletal muscle macrophage transcriptomes at single-cell resolution. We used a droplet-based scRNAseq platform (Chromium, 10xGenomics) to profile muscle macrophages (live F4/80^+^CD11b^+^Siglec-F^−^ cells) purified by fluorescence-activated cell sorting (FACS) from C57BL/10ScSn-Dmdmdx/J mice (B10.mdx) hindlimb muscle during the acute stage of disease (4 weeks of age) and age-matched, C57BL/10 wild-type (WT) controls (Fig. 1A). This stage was selected because muscle macrophage numbers are most elevated and their depletion reduces muscle necrosis by ~80% in mdx mice (*26*), suggesting that a peak inflammatory state is achieved by this age. FACS yielded macrophage samples with greater than 92 to 96% purity.

Uniform Manifold Approximation and Projection (UMAP) for dimensionality reduction of 11,367 single-cell profiles (WT = 5723; mdx = 5644) was used to examine the data. The resulting analysis partitioned single-cell profiles into eight clusters composed primarily of macrophages and a low proportion of contaminating cell types, including endothelial cells, FAPs, satellite cells, and muscle-like cells (fig. S1, A and B). Clusters 0, 1, 2, and 3 made up more than 90% of the single-cell profiles and were assigned a macrophage identity based on their expression of macrophage markers (fig. S1C). We filtered out the contaminating cell types and reclustered the single-cell profiles corresponding to clusters 0 to 3 in fig. S1A, which resulted in the identification of six macrophage clusters (fig. S1D).

The proportion of cluster 0 to 5 macrophages differed among healthy and dystrophic muscle (Fig. 1, B and C). Cluster 1 macrophages were almost exclusively present in healthy muscle, whereas clusters 0 and 2 were predominantly found in dystrophic muscle. Clusters 3 to 5 were shared between healthy and dystrophic muscle. Vast differences in transcriptional profiles were noted between macrophages isolated from dystrophic (red) and WT, healthy muscle (blue) (Fig. 1D). Hierarchical cluster analysis of differentially expressed genes (DEGs) revealed that each macrophage population expressed distinct transcriptional modules containing cluster-specific genes with biomarker potential (Fig. 1E).

Cluster 1 macrophages were characterized by increased expression of *Folr2*, *Mt2*, *Lyve1*, *Gas6*, and *Cbr2*. This signature shared similarities with a subset of skeletal muscle–resident macrophages (SkMRMs) previously described in healthy muscle (*27*). Hereafter, cluster 1 macrophages are referred to as SkMRMs. Cluster 0 macrophages (hereafter referred to as gal-3^+^ macrophages) expressed high levels of *Spp1*, *Fabp5*, *Gpnmb*, *Trem2*, *Lgals3*, and various cathepsin genes. *Lgals3* (gal-3) has been implicated in the development of fibrosis (*28*, *29*), suggesting that gal-3^+^ macrophages promote fibrosis during muscular dystrophy. In support of this, *Spp1* (osteopontin) also promotes muscle fibrosis in mdx mice (*22*) through a matrix metalloproteinase-mediated processing of TGF-β in stromal cells (*30*).

Cluster 2 macrophages, hereafter referred to as MDMs, were marked by *Cd52*, *Plac8*, *Prdx5*, and *Hp*. F4/80 (*Adgre1*), which is lowly expressed in blood monocytes (fig. S2A) (*31*), was expressed lower in MDMs relative to SkMRMs or gal-3^+^ macrophages (fig. S2B). *Ly6c2* and *Cd52*, which are highly expressed in blood monocytes (fig. S2, C and E), were also highly expressed in MDMs, clusters 3 and 5 (fig. S2, D and F). Flow cytometry analysis of healthy and dystrophic muscle macrophages confirmed these observations (fig. S2, G to J). Further, MDMs expressed high levels of *Ccr2* but low levels of *Cx3cr1* (fig. S2, K and L). Collectively, these findings suggest that cluster 2 is a monocyte-derived population that resembles Ly6c^hi^CCR2^+^ inflammatory monocytes.

Cluster 3 was defined by high expression of *Cd74*, major histocompatibility complex II genes (*H2-Eb1*, *H2-DMb2*, *H2-Ab1*, *H2-Aa*, *H2-DMb1*, and *H2-DMa*) and the dendritic cells marker *Itagx*, suggesting that this population corresponds to dendritic cells (Fig. 1E and fig. S2M). Cluster 4 macrophages expressed genes associated with chromatin, nucleosome (*H2afv*, *Top2a*, *Hmgb2*, and *Hmgn2*), and cell cycle (*Birc5*, *Cks1b*, and *Cdca3*) regulation. Cluster 5 defined a macrophage population characterized by the high expression of genes associated with interferon signaling (*Irf7*, *Ifi47*, *Ifit3*, *ifit2*, and *Isg20*).

### Isolation and bulk transcriptome profiling of SkMRMs, MDMs, and gal-3^+^ muscle macrophages by bulk RNAseq

A flow cytometry panel was developed to validate the scRNAseq macrophage populations that were predominantly associated with either homeostasis or dystrophinopathy. Gal-3, Folr2, and CD52, which were preferentially expressed by clusters 0, 1 and 2, respectively, were used as markers to distinguish gal-3^+^ macrophages, SkMRMs and MDMs (fig. S3, A and B). Macrophages in WT muscle did not express gal-3 but highly expressed *Folr2*, similar to SkMRMs identified in the scRNAseq analysis (fig. S3, A and B). In healthy tissues, Folr2^+^ macrophages were most abundant in skeletal muscle (72% ±2.5) followed by the heart (30% ±3.4) (fig. S4) but nearly absent in the brain, bone marrow, and blood. This expression pattern expands on the observations made in an earlier study examining Folr2 in tissue-resident macrophages (*32*). A gal-3^hi^Folr2^lo^ macrophage population was also identified in dystrophic muscle that corresponded to gal-3^+^ macrophages in the scRNAseq (fig. S3, A and B), which was largely absent in several healthy tissues (fig. S4). CD52 was most highly expressed in gal-3^−^Folr2^−^ (WT) or gal-3^lo^Folr2^−^ (mdx) macrophages and marked a population that likely corresponded to the MDMs in the scRNAseq analysis (fig. S3, A and B). CD52^+^ monocytes or macrophages were present in all WT tissues examined with the highest proportion in the blood, lung, liver, and adipose tissue (fig. S4). Collectively, the flow cytometry panel discriminated three muscle macrophage populations based on gal-3, Folr2, and CD52 expression.

**Fig. 2.**
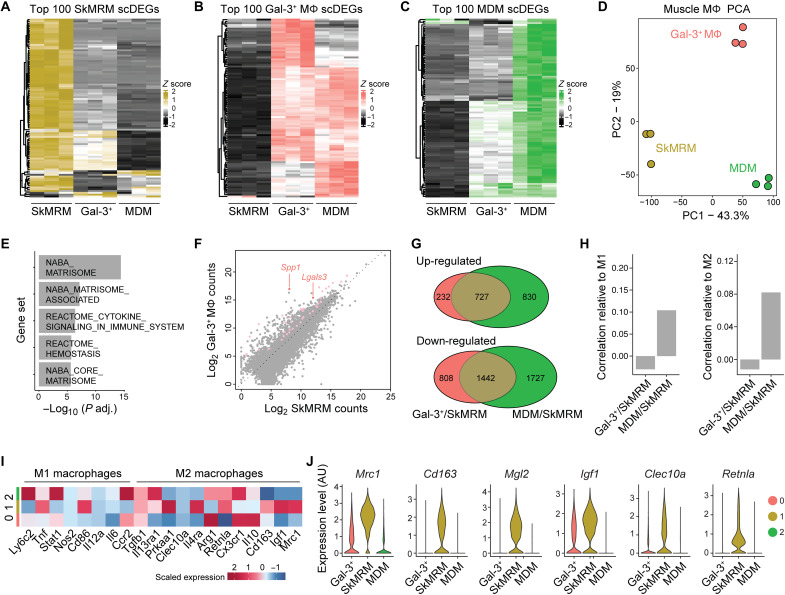
Muscle macrophages express transcriptomes distinct from M1 and M2 macrophages. (**A** to **C**) Heatmap showing the expression of the top 100 scRNAseq DEGs from the scRNAseq analysis (scDEGs) in FACS-sorted SkMRM (A), gal-3^+^ Mϕ (B), and MDM (C). *n* = 3 per population. (**D**) PCA applied to the FACS-sorted macrophage populations in (A) to (C). (**E**) Pathway analysis of top gene sets enriched in FACS-sorted gal-3^+^ Mϕ compared to SkMRM. (**F**) Pairwise comparison of gene expression between FACS-sorted gal-3^+^ Mϕ and SkMRM. Colored points indicate DEGs from ECM-related gene sets. *Lgals3* and *Spp1* are highlighted by arrows. (**G**) Venn diagrams of up-regulated and down-regulated DEGs in FACS-sorted gal-3^+^ Mϕ compared to SkMRM (Gal-3^+^/SkMRM) and MDMs compared to SkMRMs (MDMs/SkMRMs). (**H**) Correlation analysis of DEGs from Gal-3^+^/SkMRM or MDM/SkMRM comparisons with M1 or M2 polarized Mϕ. (**I**) Expression of M1 and M2 macrophage markers in gal-3^+^ Mϕ (0), SkMRMs (1), and MDMs (2) from scRNAseq data. (**J**) Violin plots of M2 markers in gal-3^+^ Mϕ, SkMRMs, and MDMs from the scRNAseq data. AU, arbitrary units.

To further link the flow cytometry macrophage populations to the scRNAseq profiles and establish their full transcriptomes, we performed bulk RNAseq on FACS-sorted populations. The macrophage populations were sorted from 4-week-old WT (SkMRM) and dystrophic hindlimb muscle (gal-3^hi^Folr2^lo^ and MDMs). The top 100 scRNAseq differentially expressed genes (scDEGs) from each cluster were used to match transcriptomes to the sorted populations. The SkMRM, gal-3^+^, and MDM scDEGs nearly uniformly distinguished the three transcriptomes and confirmed their match to the single-cell populations (Fig. 2, A to C, and fig. S3, C to E). In addition, the gal-3^+^ scDEGs indicated partial overlapping profiles with the MDM scDEGs suggesting a cell state transition (Fig. 2B). Consistent with this, the gal-3^+^ population down-regulated resident (*Folr2*, *Gas6*, *Cbr2*, and *Lyve1*) and MDM (*Cd52*, *Ccr2*, and *Ly6c2*) marker genes (fig. S3F). These observations suggest that the gal-3^+^ population is a terminal transition state of either resident or MDMs.

To further probe the macrophage transcriptomes, we performed principal components analysis (PCA) and classified DEGs. Using the SkMRM dataset and publicly available microglia and bone marrow–derived macrophage (BMDMϕ) datasets (*33*, *34*), the analysis showed that ECM and development genes were enriched in SkMRMs, suggesting a role in muscle homeostasis (fig. S5). The PCA was next used to classify the variation between the sorted gal-3^+^ macrophages, MDMs, and SkMRMs (Fig 2D). The largest variance (PC1) separated normal and dystrophic macrophages and contained genes enriched in the innate immune response (fig. S3G). The second largest (PC2) variation was enriched for genes in pathways associated with the lysosome and lipid metabolism, suggesting that gal-3^+^ macrophages exhibit enhanced phagocytosis of muscle debris and lipid membranes (fig. S3H). By directly comparing DEGs between the gal-3^+^ macrophages and SkMRMs, ECM and inflammation-related pathways were further identified as highly altered by the dystrophic environment (Fig. 2E). We determined 959 and 2250 up-regulated and down-regulated genes, respectively, in the gal-3^+^ macrophages compared to SkMRMs (fig. S3I and table S1). Consistent with marker gene up-regulation, *Lgals3* and *Spp1* were among the highest up-regulated and expressed genes in the gal-3^+^ state (Fig. 2F).

The common transcriptional response of gal-3^+^ macrophages and MDMs to the dystrophic environment was indicated by an overlap of 727 up-regulated (41% of total) and 1442 down-regulated (36% of total) DEGs compared to SkMRMs (Fig. 2G, *P* values > 1 × 10^−16^). Pathways enriched within the common DEGs indicated up-regulated genes involved in leukocyte activation and the inflammatory response and down-regulated genes in glycosaminoglycan and ECM-related pathways (fig. S3J). Gal-3^+^ macrophages and MDMs also expressed unique DEGs, indicating specialized functional states for distinct dystrophic muscle macrophage populations. The analysis of enriched pathways in the unique DEGs again indicated ECM and lysosomal gene up-regulation in the gal-3^+^ state compared to the MDMs, potentially reflecting enhanced fibrotic and phagocytic activity (fig. S3, K and L).

To further characterize the inflammatory states of the dystrophic macrophages, we compared them to well-defined M1 and M2 polarization signatures established by in vitro treatments of BMDMϕ (*34*). The transcriptional profiles substantially differed between muscle and BMDMϕ macrophages (fig. S3M), and little to no correlation was found between the MDM (C2/C1) or gal-3^+^ state (C0/C1) and M1 or M2 polarization states (Fig. 2H). Further, several M1 and M2 markers were heterogeneously expressed across the dystrophic macrophage populations in the scRNAseq analysis (Fig. 2I). Unexpectedly, some M2 markers were expressed highest in SkMRMs (Fig. 2J). Flow cytometry and quantitative polymerase chain reaction (qPCR) analysis confirmed that M1 and M2 genes were not selectively enriched in any of the scRNAseq-defined muscle macrophage populations (fig. S6). The transcriptomic profiling of muscle macrophages supports that a terminal differentiation state marked by high gal-3 expression is a dominant signature induced in muscular dystrophy. This gal-3 signature is associated with regulation of the ECM and has little overlap with M1 and M2 macrophage activation signatures.

### Spatial transcriptomic analysis reveals that gal-3^+^ macrophages are associated with stromal cells and ECM genes

**Fig. 3.**
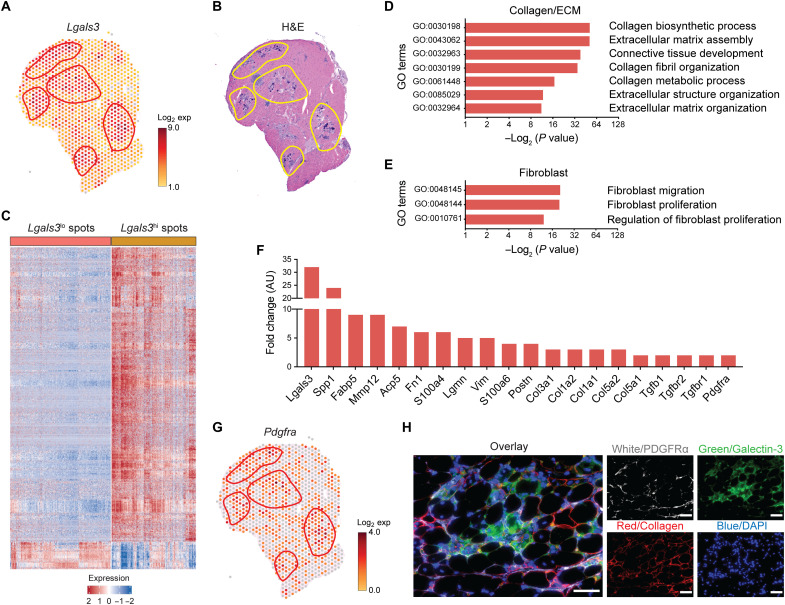
Spatial transcriptomics reveals that gal-3^+^ macrophages are associated with stromal cells and ECM. (**A** and **B**) Spatially resolved gene expression of *Lgals3* (gal-3) (A) and hematoxylin and eosin (H&E) staining of D2-mdx quadriceps. (B) Shown is one of five representative D2-mdx quadriceps. (**C**) Heatmap showing DEGs between Lgals3^hi^ and Lgals3^lo^ spots. Shown are genes with a fold change ≥ 1.5 and false discovery rate < 0.01. All spots in a section, including those with and without pathology, were unbiasedly analyzed. (**D** and **E**) Gene ontology (GO)/pathway analysis showing the enrichment of GO terms in gal-3^hi^ spots associated with collagen/ECM (D) and fibroblasts (E). (**F**) Expression of DEGs associated with fibrosis. (**G**) Spatially resolved gene expression of *Pdgfra* in mdx quadriceps. (**H**) Immunofluorescence staining of 4-week-old mdx quadriceps with anti-PDGFRα (white), anticollagen (red), and anti–gal-3 (green) antibodies. Scale bars, 50 μm. DAPI, 4′,6-diamidino-2-phenylindole.

We used capture probe–based spatial transcriptomics (Visium, 10x Genomics) to gain insight on how gal-3^+^ macrophages functionally and spatially interfaced with the dystrophic environment. Spatial RNA sequencing was performed on the gastrocnemius/plantaris muscle complex of 6-week-old mdx mice in the DBA2/J background (D2-mdx). This approach provides spatially resolved gene expression analysis limited in resolution by 55-μm-diameter spot size. Interrogation of the spatial matrix revealed that areas with high gal-3 expression (Fig. 3A, highlighted in red) were confined to regions with active pathology that were densely populated by mononuclear cells (Fig. 3B, highlighted in yellow).

A differential gene expression analysis between gal-3^hi^ and gal-3^lo^ spots revealed that 1365 and 125 genes were up- and down-regulated, respectively (Fig. 3C). The provided heatmap comprises 569 gal-3^hi^ and 676 gal-3^lo^ spots collected from five D2-mdx gastrocnemius/plantaris muscle complexes. We assigned any spot with a unique molecular identifier (UMI) count ≥ 3 as gal-3^hi^ and spots with a UMI count ≤ 1 as gal-3^lo^. A gene ontology (GO) analysis was performed on the DEGs, which showed that phagocytosis, endocytosis, and leukocyte migration were among the most enriched terms (fig. S7A). Terms associated with regeneration and repair were also highly enriched in the gal-3^hi^ areas (fig. S7B). Of particular interest, GO terms associated with the ECM and fibroblasts (Fig. 3, D and E) and multiple genes associated with fibrosis were enriched in the gal-3^hi^ areas (Fig. 3F). *Lgals3* and *Spp1* were among the highest, revealing a greater than 30- and 25-fold increase, respectively. The profibrotic matrix metalloproteinase-12 (*Mmp12*) was also increased (*35*). Genes encoding ECM components were increased, including fibronectin (*Fn1*), periostin (*Postn*), and several collagens, as well as components of growth factor pathways that induce fibrosis (e.g., *Tgf*β *and Pdgf*).

The spatial transcriptomics also revealed that platelet-derived growth factor receptor–α (PDGFRα), a growth factor receptor expressed on stromal cells, was increased in the gal-3^+^ areas (Fig. 3G, highlighted in red). Immunofluorescence assays performed on mdx quadriceps revealed that PDGFRα^+^ stromal cells in pathological lesions were juxtaposed with gal-3^+^ macrophages (Fig. 3H). Collectively, these findings suggest that gal-3^+^ macrophages interact with stromal cells (e.g., FAPs) in degenerative lesions to promote fibrosis.

### Intercellular communication network analysis identifies that FAPs and macrophages communicate via Spp1

**Fig. 4.**
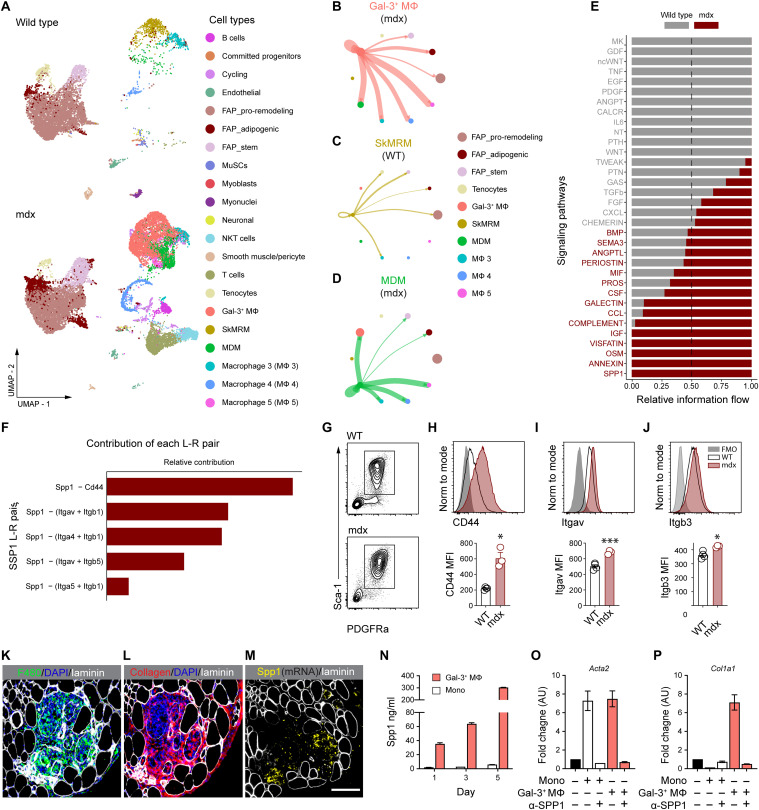
Spp1 mediates FAP and macrophage interactions in dystrophic muscle. (**A**) Reference-based integration of skeletal muscle mononucleated cell datasets prepared from 3-month-old WT and mdx mice. (**B** to **D**) Visualization and analysis of cell-cell communication using CellChat. Circle plots placing macrophage subsets as the central nodes of analysis in the mdx dataset (B) to (D). An interaction between a pair of cell types is depicted by a line connecting two cell types. The thickness of the line depicts the strength of that interaction. (**E**) Pathways enriched in the stromal cell and macrophage network of WT and mdx mice. (**F**) Relative contribution of Spp1 ligand (L)–receptor (R) pairs. (**G**) Expression of Spp1 receptors was measured in WT and mdx PDGFRα^+^Sca1^+^ FAPs by flow cytometry. Plots shown were gated on live CD45^−^CD31^−^ cells. (**H** to **J**) Representative histograms and quantification of the mean fluorescence intensity (MFI) of Spp1 receptors. Four-week-old mice were analyzed. *n* = 3 to 4. *P < 0.05 and ***P < 0.001 using an unpaired Welch’s *t* test. (**K** to **M**) RNAscope multiplexed with immunofluorescence staining of adjacent section showing F4/80^+^ macrophages (K, green), *Spp1* mRNA (M, yellow), and areas enriched with collagen (L, red). Laminin is shown in white (K) to (M). Scale bar, 100 μm. (**N**) Spp1 secretion was assessed by enzyme-linked immunosorbent assay. (**O** and **P**) The expression of *Acta2* (O) and *Col1a1* (P) was measured by reverse transcription qPCR (RT-qPCR) in FAPs stimulated with monocyte (mono)–conditioned or gal-3^+^ macrophage–conditioned media. α-SPP1, neutralizing mouse Spp1 antibody. Shown is a representative of two independent experiments with conditions done in duplicate.

To determine how gal-3^+^ macrophages and stromal progenitors interact, we performed a reference-based integration of skeletal muscle mononucleated cell datasets and surveyed intercellular communication networks with CellChat (*36*). We used a publicly available dataset of uninjured muscle (*37*) and referenced an additional dataset independently prepared from muscle mononucleated cells pooled from three mdx mice. Three subpopulations of FAPs, including adipogenic, proremodeling, and stem populations, were identified, as reported previously (*38*), in uninjured and dystrophic muscle. An increased proportion of proremodeling (53.2% versus 21.6%) and adipogenic FAPs (8.67% versus 5.70%) was noted in dystrophic muscle, as well as an overall expansion of muscle macrophages (38.9% versus 14.4%), compared to healthy muscle (Fig. 4A). Similar to the scRNAseq analysis of purified macrophages (Fig. 1), gal-3^+^ macrophages were the predominant macrophage population in dystrophic muscle (Fig. 4A). The probability of communication between two cell groups was visualized with circle plots by designating FAPs as the central nodes of analysis. The highest degree of inferred communication occurred with gal-3^+^ macrophages, reflected by the thickness of the connecting edge (fig. S8, A to C). The CellChat analysis was also performed with gal-3^+^ macrophages, SkMRM, or MDMs as the central nodes of analysis. Circle plots show a large probability of intermacrophage communication, followed by communication with FAP populations (Fig. 4, B to D). Little to no communication was found between macrophages and tenocytes.

Next, information flow for signaling pathways in the stromal cell and macrophage network was assessed (Fig. 4E). Some pathways (e.g., CXCL, CHEMERIN, BMP, SEMA3, ANGPTL, and PERIOSTIN) maintain similar flow in healthy and dystrophic muscle, suggesting that these pathways are equally important in regulating macrophage and FAP interaction during homeostasis and muscle disease. In contrast, other pathways prominently change their information flow in dystrophic muscle compared to uninjured muscle. For example, MK and growth differentiation factor (GDF) are silenced in dystrophic muscle, and TWEAK, PTN, and GAS are decreased. In contrast, GALECTIN, CCL and COMPLEMENT are increased in dystrophic muscle. Insulin-like growth factor (IGF), VISFATIN, OSM, ANNEXIN and SPP1 were exclusively active in dystrophic muscle.

The Spp1 pathway was further characterized given its known role in promoting muscle fibrosis during muscular dystrophy (*22*). The highest relative contribution to the Spp1 pathway was attributed to the Spp1-CD44 ligand (L)–receptor (R) pair, followed by several integrin heterodimer receptors (Fig. 4F). Flow cytometry showed that Spp1 receptors—CD44, Itgav, and Itgb3—were up-regulated in mdx PDGFRα^+^Sca1^+^ stromal cells, compared to WT stromal cells (Fig. 4, G to J). RNAscope multiplexed with immunofluorescence staining revealed that degenerative lesions with increased macrophages and collagen contained elevated levels of *Spp1* (Fig. 4, K to M). In vitro culturing of gal-3^+^ muscle macrophages revealed that they secreted higher levels of Spp1 compared to bone marrow monocytes (Fig. 4N). Monocyte- and gal-3^+^ macrophage–conditioned media induced the expression of *Acta2* in FAPs in a Spp1-dependent manner, but only gal-3^+^ macrophage–conditioned media increased the expression of *Col1a1* (Fig. 4, O and P). Collectively, these results suggest that the Spp1 pathway is a key regulator of gal-3^+^ macrophage and FAP interactions during muscular dystrophy and that Spp1 signals primarily through CD44 and a subset of integrin heterodimers to control FAP differentiation.

### Muscle damage expands a population of gal-3^+^ macrophages that is chronically activated in muscular dystrophy

**Fig. 5.**
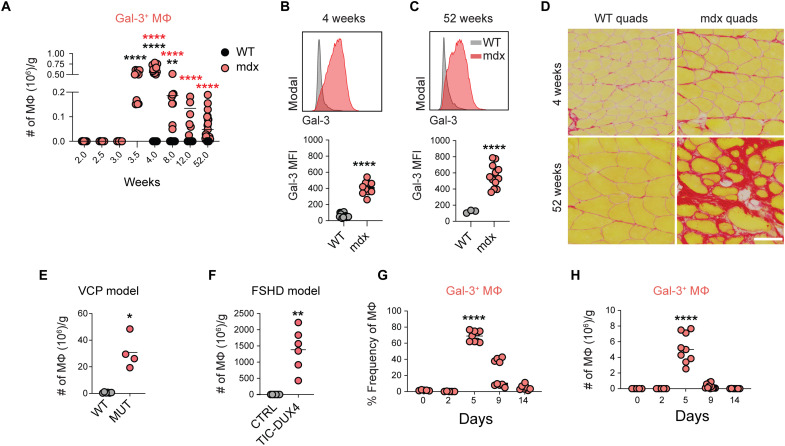
Chronic activation of gal-3^+^ macrophages in dystrophic muscle. (**A**) The number of gal-3^+^ macrophages in B10.mdx hindlimb muscle, normalized to muscle mass (grams). *n* = 6 to 19 per time point. (**B** and **C**) Representative histograms and quantitative analysis of the geometric MFI of gal-3 in 4-week-old (B) and 52-week-old (C) WT and mdx muscle macrophages. (**D**) Picrosirius red staining of WT and mdx quadriceps cryosections from 4- and 52-week-old mice. Scale bar, 100 μm. (**E** and **F**) Enumeration of gal-3^+^ macrophages in the VCP-associated inclusion body myopathy mouse model (E) and in the facioscapulohumeral muscular dystrophy Tamoxifen inducible Cre-DUX4 (TIC-DUX4) mouse model (F). *n* = 4 to 6, 10-month-old mice (E); *n* = 5, 10-week-old mice (F). (**G** and **H**) Regulation of gal-3^+^ macrophages frequency (G) and number (H) after injury. *n* = 7 to 9 per time point (G) and (H). *P < 0.05, **P < 0.01, and ****P < 0.0001 using an unpaired Welch’s *t* test (B), (C), (E), and (F) or two-way ANOVA with Sidak’s multiple comparisons test, for comparison with 2-week time point (A) or with day 0 (G) and (H). ****P < 0.0001 using Sidak’s multiple comparisons test, for comparison with age-matched controls of 4, 8, 12, and 52 weeks, respectively.

We next examined the regulation of muscle macrophage populations during muscular dystrophy. SkMRM and MDMs were elevated in the early stages of disease but resolved by chronic stages (fig. S9, A and B). An elevation of gal-3^+^ (gal-3^hi^Folr2^lo^) macrophages was observed as early as 3.5 weeks of age in mdx hindlimb muscles and began to decline by 8 weeks but remained chronically elevated up to 52 weeks of age compared to controls (Fig. 5A). Gal-3 protein was up-regulated in dystrophic muscle macrophages as early as 4 weeks and remained elevated at 52 weeks of age (Fig. 5, B and C). Immunofluorescence staining showed that gal-3 was expressed in a subset of F4/80^+^ macrophages in dystrophic muscle but absent in WT muscle macrophages (fig. S9C). The chronic activation of gal-3^+^ macrophages was associated with increased collagen deposition at 52 weeks (Fig. 5D).

The induction of a gal-3^+^ transcriptional program was conserved in other forms of muscle disease. We performed a referenced-based integration of muscle macrophage scRNAseq datasets prepared from 8-month-old B6A/J mice, a mouse model of limb girdle muscular dystrophy 2B, and respective controls (fig. S10A). The mdx dataset from this study (Fig. 1) was used as the reference. Similar to mdx dystrophic muscle, the predominant muscle macrophage population in B6A/J mice corresponded to gal-3^+^ macrophages (fig. S10B). Immunofluorescence staining of 12-month-old muscle confirmed the presence of gal-3^+^ macrophages and their proximity to PDGFRα^+^ stromal cells in B6A/J mice (fig. S10D). Gal-3^+^ macrophages were largely absent in B6 healthy control muscle (fig. S10C). SkMRMs were similarly the dominant macrophage population in control muscle. Although the lack of MDMs and cluster 5 macrophages in B6A/J and control samples could be explained by disease-specific differences, we cannot rule out that differences in the stage of disease (i.e., age) could contribute to this observation. Gal-3^+^ muscle macrophages were also elevated in mouse models of valosin-containing protein (VCP)–associated inclusion body myopathy and facioscapulohumeral muscular dystrophy (Fig. 5, E and F).

The regulation of gal-3^+^ macrophages following BaCl_2_-induced acute injury was also examined. A large increase in the proportion and number of gal-3^+^ muscle macrophages occurred 5 days after injury (Fig. 5, G and H). By day 9, gal-3^+^ macrophages began to contract and returned to their homeostatic levels by day 14. The expansion of gal-3^+^ macrophages at day 5 coincided with the transition of muscle toward the repair phase and when collagen deposition was most apparent, suggesting that they are involved in remodeling of the ECM during muscle regeneration (fig. S11, A and B).

### Gal-3^+^ macrophages are derived from SkMRMs and peripheral monocytes

**Fig. 6.**
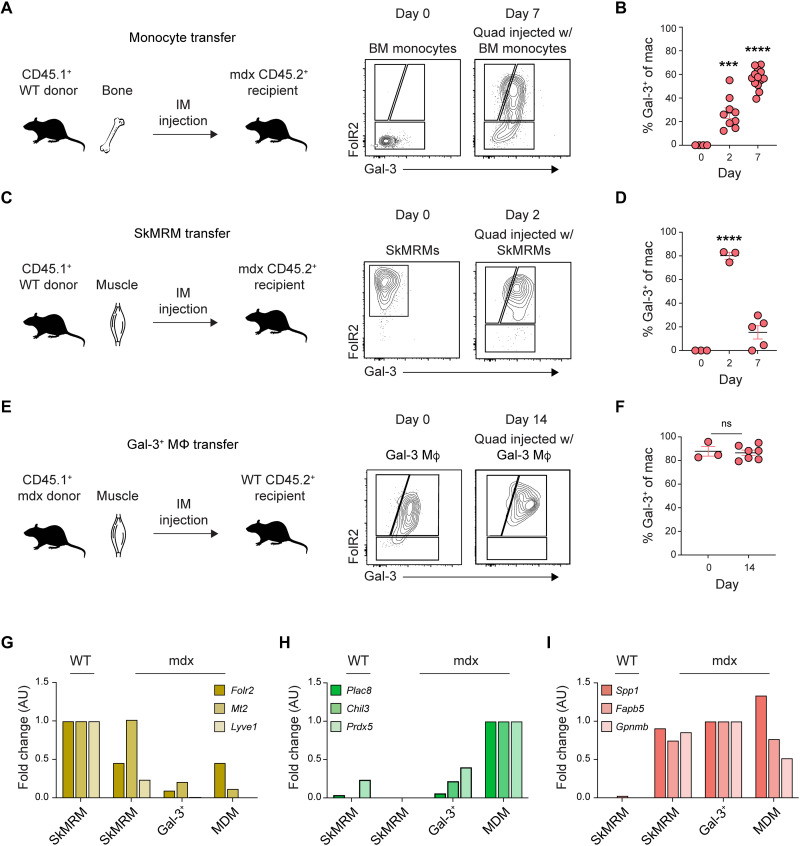
Peripheral monocytes and skeletal muscle-resident macrophages give rise to gal-3^+^ macrophages. (**A** and **B**) Adoptive transfer of monocytes into 4-week-old mdx mice. Graphical abstract of the workflow and representative flow plots of monocytes before and after transfer (A). Frequency of the donor monocytes that converted to gal-3^+^ macrophages at 2 and 7 days after transfer (B). *n* = 3 to 13 per group. (**C** and **D**) Adoptive transfer of SkMRMs into 4-week-old mdx mice. Schematic of the workflow and representative flow plots (C). Frequency of SkMRMs that converted to gal-3^+^ macrophages (D). *n* = 3 to 5 per group. (**E** and **F**) Schematic of the transfer of gal-3^+^ macrophages into healthy WT muscle (E) and their quantification (F). *n* = 3 to 7 per group. (**G** to **I**) RT-qPCR quantification of the expression of cluster 1 (E), 2 (F), and 0 genes (G) in FACS-sorted SkMRMs from WT and mdx muscle, and gal-3^+^ Mϕ and MDMs from mdx muscle. ***P < 0.001, and ****P < 0.0001 using a one-way ANOVA with Sidak’s multiple comparisons test (B) and (D) for comparison with the day 0 mean or an unpaired Welch’s *t* test (F). A two-way ANOVA with Sidak’s multiple comparisons test was used for the gene expression assays (G) to (I). IM, intramuscular; ns, not significant.

The capacity of SkMRMs and monocytes to differentiate into gal-3^hi^Folr2^lo^ macrophages was determined through adoptive transfer assays. SkMRMs or bone marrow monocytes from CD45.1^+^ congenic WT mice were injected into the quadriceps of 4-week-old mdx.CD45.2^+^-recipient mice. Before the adoptive transfer (day 0), bone marrow monocytes did not express Folr2 or gal-3 (Fig. 6A). CD45.1^+^-transferred monocytes up-regulated gal-3 and Folr2, leading to an increased proportion of cells that acquired the gal-3^hi^Folr2^lo^ phenotype as early as day 2 and remained elevated 7 days after transfer (Fig. 6, A and B). The adoptive transfer of CD45.1^+^ SkMRMs from WT muscle resulted in a down-regulation of Folr2 and an up-regulation of gal-3, resembling the gal-3^hi^Folr2^lo^ phenotype by the second day after transfer (Fig. 6, C and D). CD45.1^+^ SkMRMs were substantially declined 7 days after transfer, suggesting that gal-3^hi^Folr2^lo^ emerging from the SkMRM pool are short-lived (Fig. 6D). Intriguingly, mdx gal-3^+^ macrophages transferred into healthy WT muscle did not revert to a SkMRM phenotype, suggesting that they exhibit little plasticity in this setting (Fig. 6, E and F). Despite their inability to resolve, gal-3^+^ macrophages did not promote collagen deposition in WT muscle or induce the expression of genes associated with muscle fibrosis (fig. S12) (*39*–*41*).

The regulation of SkMRMs, MDMs, and gal-3^+^ macrophage marker genes in endogenous macrophages FACS-sorted from 4-week-old WT and mdx muscle was assessed by reverse transcription qPCR (RT-qPCR) (Fig. 6, H and I). The following marker genes were interrogated: *spp1*, *Fabp5*, and *Gpnmb*; *Folr2*, *Mt2*, and *Lyve1*; and *Plac8*, *Chil3*, and *Prdx5*, because of their preferential expression in gal-3^+^ macrophages, SkMRMs, and MDMs, respectively (fig. S13). Folr2^hi^ macrophages isolated from dystrophic muscle (mdx SkMRM) began to lose expression of SkMRM genes (Fig. 6G) but gained expression of gal-3^+^ macrophage genes (Fig. 6I). Similarly, MDMs in dystrophic muscle up-regulated gal-3^+^ macrophage genes (Fig. 6I), whereas MDM genes were lowly expressed in gal-3^+^ macrophages (Fig. 6H). This regulation of SkMRM, MDM, and gal-3^+^ macrophage genes is consistent with the adoptive transfer assays and supports the interpretation that SkMRMs and recruited CD52^+^ monocytes are activated and differentiate into gal-3^hi^Folr2^lo^ macrophages within the dystrophic niche.

### Gal-3^+^ macrophages are elevated in human diseased muscle

**Fig. 7.**
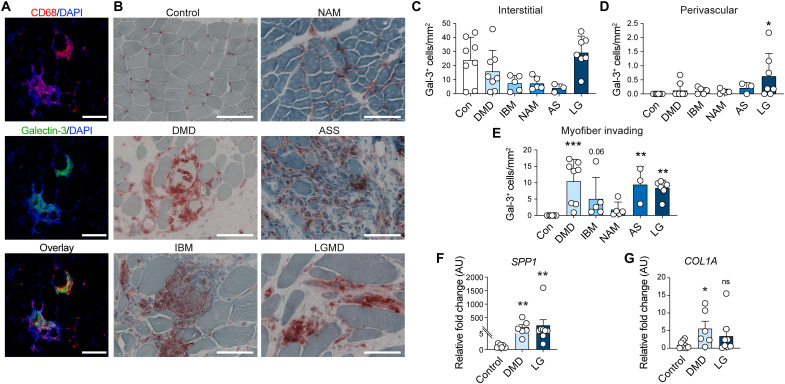
Gal-3^+^ macrophages are elevated in human chronic muscle disease. (**A**) Immunofluorescence staining of gal-3^+^ macrophages in human IBM muscle. CD68 (red), gal-3 (green), nuclei (blue). Scale bars, 100 μm. (**B**) Representative images of immunohistochemical staining of gal-3 in control and myopathic patients. Scale bars, 100 μm. (**C** to **E**) Quantification of gal-3^+^ cells in the interstitial space (C), the perivascular area (D), or infiltrating the myofiber (E). *n* = 3 to 8 frozen sections per patient type. (**F** and **G**) Expression of human *SPP1* (F) and *COL1A* (G) mRNA in control, DMD, and LGMD biopsies. *n* = 6 to 8 patients were used to measure RNA expression. *P < 0.05, **P < 0.01, and ***P < 0.001 using a two-way ANOVA with Kruskal-Wallis multiple comparisons test (C) to (G).

To determine whether gal-3^+^ macrophages are also present in human dystrophic muscle, we quantified their numbers in archived muscle biopsies. Immunofluorescence assays showed that gal-3 was expressed in a subset of CD68^+^ macrophages (Fig. 7A). An immunohistochemical examination of gal-3 showed that the number of gal-3^+^ macrophages in interstitial or perivascular regions did not differ between control and myopathic patients, except for an increase in gal-3^+^ perivascular macrophages in limb girdle muscular dystrophy 2A (LGMD2A) (Fig. 7, B to D). However, the number of gal-3^+^ myofiber-invading macrophages was significantly elevated in DMD, antisynthetase syndrome (ASS), and LGMD2A (Fig. 7, B and E). A nonsignificant trend for an increase in patients with inclusion body myositis (IBM) was also noted. Consistent with the increase in gal-3^+^ macrophages in DMD and LGMD2A, the expression of *SPP1* in whole muscle was elevated (Fig. 7F). RNA was not available for IBM, necrotizing autoimmune myopathy (NAM), and ASS groups to measure transcript levels of *SPP1*. Further, *COL1A* mRNA was significantly elevated in DMD muscle, suggesting that a similar gal-3^+^ macrophage and Spp1 pathway promotes fibrosis in DMD (Fig. 7, F and G).

## DISCUSSION

Macrophage diversity is regulated by the integration of tissue-intrinsic cues with inflammatory signals to induce unique transcriptional programs. Single-cell transcriptomics has helped uncover an unprecedented degree of macrophage heterogeneity attributed to the integration of these complex signals in several organs, including the heart, brain, and adipose tissue (*42*–*44*). Recently, comparative single-cell transcriptomic studies of tissue-resident macrophages isolated from distinct anatomical sites, including skeletal muscle, demonstrated that muscle macrophages expressed a transcriptome that differs from those of lung and peritoneal macrophages (*27*). Transcriptomic analysis of muscle macrophages isolated from acutely injured muscle has also shown that macrophages acquire distinct transcriptional programs adapted to promoting muscle regeneration (*45*, *46*). However, the diversity of muscle macrophages and how their function and transcriptional states are influenced by muscle disease in an immunocompetent setting have not been defined.

Here, we performed an unbiased scRNAseq analysis of muscle macrophages from healthy and dystrophic muscle to define the transcriptional programs induced in macrophages that promote muscle fibrosis during muscular dystrophy. We found six distinct macrophage populations, none of which resembled polarized M1 or M2 macrophages. Rather, the six populations expressed varying degrees of M1 and M2 macrophage marker genes. The gal-3^hi^Folr2^lo^ (gal-3^+^) signature emerged as the predominant transcriptional state in dystrophic muscle macrophages. Gal-3^+^ macrophages expressed profibrotic factors, including Spp1 and gal-3, and were chronically activated in muscular dystrophy. A recent scRNAseq analysis of immunodeficient mdx mice (mdxNSG) described a M2c-like macrophage population that was marked by high expression of *Spp1*, suggesting that adaptive immunity likely has a minimal role in the induction of the gal-3^+^/Spp1^+^ macrophage state (*47*). Gal-3^+^ macrophages interacted with stromal cells (e.g., FAPs) in regions enriched with genes associated with muscle fibrosis, and computational inferences predicted that communication between these cells is partly mediated by Spp1. Gal-3^+^ macrophages were also elevated in several human myopathies, including DMD, and were juxtaposed with PDGFRα^+^ stromal cells. Further, *SPP1* and collagen were up-regulated in DMD biopsies, suggesting that a Spp1-mediated gal-3^+^ macrophage and stromal cell interaction is conserved in human to promote muscle fibrosis.

Consistent with a potential role for gal-3^+^ macrophage–derived Spp1 in the pathogenesis of muscular dystrophy, Spp1 enhances the matrix metalloproteinase 9–mediated processing of TGF-β into its active form to promote muscle fibrosis in mdx mice (*22*, *23*, *30*). Further, Spp1 skews macrophages toward a proinflammatory phenotype, which promote muscle damage in acute stages of disease in mdx mice (*10*, *23*). Spp1 and gal-3 are also associated with tissue fibrosis (*48*–*52*) and adipogenic differentiation of FAPs in skeletal muscle (*53*), suggesting a cooperative role for gal-3 and Spp1 in inducing fatty fibrosis in DMD. In support of this, Spp1 and gal-3 induce fibroblast proliferation and differentiation of myofibroblasts (*54*, *55*). It is essential to note, however, that gal-3 may have a potential role in repair as demonstrated by the emergence of gal-3^+^ macrophages in damaged tissues (*56*–*58*) and the impairment of tissue repair in gal-3–deficient mice (*59*, *60*). Studies of barium chloride–induced muscle injury or autologous muscle grafting in mice showed that gal-3 and Spp1 promote regeneration in acute settings (*61*–*63*). These findings suggest that muscle regeneration and fibrosis share common pathways of regulation, and a temporal-dependent mechanism partly determines whether Spp1 and gal-3 promote regeneration or fibrosis. This perspective is consistent with the view that fibrosis, to some extent, reflects a dysregulated repair process (*7*, *64*). While experimental approaches targeting a single factor (e.g., Spp1 or gal-3) will advance our knowledge on how they individually contribute to muscle fibrosis, resetting the transcriptional program of gal-3^+^ macrophages to its homeostatic state is likely a more effective therapeutic strategy for DMD.

In this regard, the present study defines the homeostatic signature of SkMRMs and establishes a posteriori knowledge to further investigate how this transcriptional state is regulated. The transcriptional profile of SkMRMs identified in this study overlapped with resident macrophages identified by scRNAseq analysis of healthy skeletal muscle (*27*, *65*). Wang and colleagues described a resident population in the diaphragm that was characterized by high expression of *Folr2*, *Lyve1*, *Ltc4s*, *Fxyd2*, and *Fcgrt*, genes that were expressed the highest in SkMRMs in the present study. Unexpectedly, we found that markers associated with M2 macrophages were most highly expressed in SkMRMs. This observation is consistent with the high expression of M2 markers in the CD209^+^ muscle–resident macrophages described by Wang *et al.* (*27*). Although SkMRMs expressed some markers of M2 macrophages, their overall transcriptional signature substantially differed from M2 macrophages polarized in vitro with interleukin-4 (IL-4) and IL-13. Given that the cytokines that induce M2 activation (e.g., IL-4 and IL-13) are increased with muscle injury or disease and are typically low or absent in healthy muscle (*39*, *66*), it is unlikely that these factors promote the M2-like phenotype of SkMRMs. Rather, tissue-associated or metabolic signals are more likely factors to induce this phenotype (*67*). Consistent with this interpretation, bulk RNAseq analysis revealed that SkMRMs were associated with ECM and muscle-associated pathways, suggesting that these homeostatic functions induce a molecular phenotype sharing some features with M2 macrophages. Although the function of SkMRMs remains to be defined, a recent study reported a population of muscle-resident macrophages marked by high expression of *Lyve1* and *Timd4* that promoted the clearance of acutely damaged muscle and promoted metabolic reprogramming of dystrophic muscle (*65*). Given that Folr2^hi^ SkMRMs also expressed high levels of *Lyve1* and *Timd4*, it is likely that these are similar populations with conserved function.

A major advancement in this study was the observation that the dystrophic environment converts muscle-resident macrophages and peripheral monocytes into gal-3^+^ macrophages. Prior studies implicated a role for recruited monocytes in the muscle pathology of mdx mice at 12 weeks of age (*68*). Although Ly6c^hi^ monocytes were reduced out to 6 months of age in CCR2-deficient dystrophic mice, myonecrosis and fibrosis returned to control levels by this point (*69*), suggesting that other monocyte or macrophage populations promote dystrophinopathy at later stages of disease. In this regard, Zhou and colleagues concluded that Ly6c^lo^ monocytes, which returned to control levels, were responsible for the lack of a sustained protective effect in CCR2-deficient dystrophic mice. Similar to Ly6c^+^ bone marrow monocytes, SkMRMs that were adoptively transferred into dystrophic muscle differentiated into gal-3^+^ macrophages, an activation state with putative fibrogenic activity. We propose that the sustained differentiation of dystrophic SkMRMs cooperates with the continuous recruitment of monocytes to promote fibrosis throughout the course of muscular dystrophy. In the absence of Ly6c^hi^ inflammatory monocyte recruitment, SkMRMs and Ly6c^lo^ monocytes become the dominant populations that promote fibrosis by adopting the gal-3^+^ program. Our observations that the transcriptional state of SkMRMs is associated with the ECM (fig. S5) and this program is retained in gal-3^+^ macrophages (fig. S3L), suggest that, provided the appropriate niche, SkMRMs are poised to differentiate into a population with an intrinsic quality to promote fibrosis. The development of lineage tracing systems to parse out the contribution of SkMRMs and MDMs to the gal-3^+^ macrophage pool and muscle fibrosis will be required in future studies.

Collectively, this study identified diverse subsets of muscle macrophages with distinct functions and transcriptional profiles. The gal-3^+^Spp1^+^ signature reflected the predominant transcriptional state of a dystrophic muscle macrophage. Colocalization of gal-3^+^ macrophages with stromal progenitors, and the observation that Spp1 mediates communication between these cell types in vitro, reinforces the importance of a macrophage-FAP fibrogenic axis in promoting the pathogenesis of muscle disease. The translational significance of this interaction is highlighted by the observation that a similar fibrogenic axis exists in human, as gal-3^+^ macrophages were elevated in several human muscle diseases. Further, gal-3^+^ macrophages were identified in three different models of chronic muscle disease and acute muscle injury, suggesting that a canonical mechanism associated with muscle damage triggers differentiation into the gal-3^+^ state. A likely candidate is phagocytosis of muscle cell debris, which has been documented as a key contributor to macrophage activation (*70*). However, this represents only one of the many macrophage-FAP fibrogenic circuits that have been previously documented in muscle (*11*, *19*). Mechanistic approaches relying on mouse genetics to study macrophage and stromal progenitor interactions will advance our understanding of how this axis promotes muscle fibrosis during muscular dystrophy. In summary, by defining the transcriptional heterogeneity of muscle macrophages, this study has advanced the understanding of macrophage activation and function during muscle homeostasis and degenerative disease. The defined transcriptional states open a path for developing groundbreaking therapeutic approaches to inhibit immune-mediated muscle fibrosis.

### Limitations of the study

Studies of acute muscle injury suggest that the functional role of FAP and gal-3^+^ macrophage interactions in muscle regeneration and fibrosis is complex. Nawaz *et al.* (*71*, *72*) demonstrated that CD206^+^ cells, presumably macrophages, inhibit the promyogenic activity of FAPs through a TGF-β–mediated repression of follistatin. TGF-β, whose processing depends on Spp1 (*30*), also promotes the fibrogenic differentiation of FAPs and fibrosis (*17*, *73*). Here, we report that CD206 is expressed in gal-3^hi^ macrophages (fig. S6D), which promoted the differentiation of FAPs in a Spp1-dependent manner. Whether gal-3^+^ macrophage–derived Spp1 promotes the processing of latent TGF-β to its bioactive form remains to be addressed. Given the lack of an in vivo functional assessment of gal-3^+^/Spp1^+^ macrophages and FAP interactions, the present findings only provide a correlative association with fibrosis and require further study. We note that a potential role for gal-3^+^ macrophages in fibrosis is further supported by studies showing that macrophages undergo a similar activation program in volumetric muscle loss (*74*).

Prior studies have shown that macrophages can also inhibit fibrosis. Macrophage depletion using CD11b–diphtheria toxin receptor (DTR) transgenic mice impairs clearance of vascular cell adhesion molecule (VCAM) positive FAPs and exacerbates collagen deposition (*75*). Because CD11b is expressed by all myeloid cells (*76*), these findings suggest that an undefined myeloid subset, distinct from gal-3^+^ macrophages, promotes clearance of VCAM^+^ FAPs to inhibit fibrosis. The enrichment of GO sets associated with ECM and developmental programs suggest that SkMRMs are a population with the functional capacity to inhibit fibrosis (fig. S5). Future studies will require specific deletion of SkMRMs to assess their role in inhibiting fibrosis.

Collectively, these findings underscore the complexity of macrophage and FAP interactions, which are regulated by cellular heterogeneity and differences in the inflammatory milieu between acutely injured versus diseased muscle. In the present study, we interrogated macrophage and FAP interactions at the acute stages of muscular dystrophy. However, the persistence of this interaction with disease progression has yet to be assessed. To elucidate the dynamics of macrophage and FAP interactions and how they regulate the progression of fibrosis, future studies will require an assessment of conditional knockouts of *Spp1* and its receptors throughout multiple stages of muscular dystrophy.

## MATERIALS AND METHODS

### Study design

This study aimed to determine how skeletal muscle macrophages promote fibrosis by using transcriptomics to define their homeostatic signature and how this state is altered with muscle disease. scRNAseq was used to define macrophage diversity in healthy and diseased muscle. Bulk RNAseq analysis was performed on the predominant macrophage populations identified in the scRNAseq studies, to determine the regulation of the transition from homeostatic to the diseased state. Spatial transcriptomics was used to understand how dystrophic macrophages interfaced with the dystrophic environment to promote fibrosis. Multiple mouse models were used to demonstrate the significance of a gal-3^+^ macrophage population in muscular disease. Further, adoptive transfer experiments were used to determine whether resident macrophages, and peripheral monocytes gave rise to gal-3^+^ macrophages. The translation of these studies was assessed by quantifying gal-3^+^ macrophages in human muscle disease through an immunohistochemical examination of archived, deidentified muscle biopsies. Prior approval for collecting muscle tissue and its use in research was given by the Institutional Review Board at the University of California Irvine (UCI; HS 2019-5134). All participants provided written informed consent and Health Insurance Portability and Accountability Act authorization for data collection and the use of muscle tissue for research.

### Experimental animals

C57BL/10 (no. 000476), mdx mice (C57BL/10ScSn-Dmdmdx/J) (no. 001801), and mdx mice in the DBA2/J background (D2-mdx) (no. 013141) were obtained from The Jackson Laboratory. CD45.1 congenic mice (no. 002014) were also obtained from The Jackson Laboratory and crossed with mdx mice at the UCI. B6A/J (no. 012767) and C57BL/6 mice (no. 000664) were bred in vivariums at Children’s National Hospital. VCP (*77*) and double homeobox 4 (DUX4) (*78*) mice were provided by collaborators at the UCI and Ohio State University, respectively. Animal experiments were approved by the Institutional Animal Care and Use Committee of UCI and performed under Institutional Animal Care and Use Committee guidelines.

### Single-cell RNA sequencing

#### Single-cell preparation and analysis of muscle macrophages

Macrophages were sorted from 4-week-old WT and B10.mdx mice, washed, and resuspended at a concentration of ~1000 cells/μl. Using the 10x Genomics platform, libraries for WT and mdx muscle macrophages were generated following the Chromium Single Cell 3′ Reagents Kits v2 User Guide: CG00052 Rev. B. Quantification of cDNA libraries was performed using the Qubit dsDNA HS Assay Kit (Life Technologies, Q32851) and high-sensitivity DNA chips (Agilent, 5067-4626). Quantification of library construction was performed using KAPA qPCR (Kapa Biosystems, KK4824). 10x Genomics libraries were sequenced on the Illumina HiSeq 4000 platform to achieve an average of ~50,000 reads per cell according to the recommendations in the Chromium Single Cell 3′ Reagents Kits v2 User Guide: CG00052 Rev. B. Sequencing reads were processed using the 10x Genomics Cell Ranger 2.1.0. Each library was aligned to an indexed mm10 genome using Cell Ranger Count. To generate an aggregated matrix of WT and mdx cells and to prepare data for downstream analysis, the Cell Ranger Aggr function was used to normalize the number of confidently mapped reads per cell in each library.

The Seurat pipeline (version 3.0.2) was applied to the aligned cell matrix using R (version 3.6.1) to identify cell clusters. Quality control filtering was first performed to remove genes that were not expressed (>0) in at least three cells and cells that had less than 200 genes. The trimmed expression count matrix was log-transformed for downstream processing, and highly variable genes were detected. PCA was combined with the elbow method to determine loadings for the generation of UMAP with 10 PCs included. Using these same PCs, Seurat’s default clustering was performed and was followed up with marker gene detection to elucidate gene expression signatures corresponding to the resultant clusters.

#### Reference-based mapping of macrophages, FAP, and tenocytes

We performed anchoring and integration of single-cell datasets of uninjured WT [i.e., day 0, publicly available dataset from Oprescu *et al.* (*37*)] and mdx mouse muscle that served as a negative control group in a separate study of mice treated with tamoxifen (dataset provided by M.J.S.). Seurat (version 3.2.2, R Studio version 3.6.1) was used for anchoring and integration (*79*). Briefly, merged Seurat objects were normalized, and highly variable genes (features) and scaling were performed with SCTransform (*80*). The top 2000 highly variable features were selected and used for anchoring. Integration anchors (30 dimensions) were computed and used for integration. For neighbor and cluster identification, the integrated object was scaled, and significant PCs were identified via statistical and heuristic testing as recommended in Seurat. Clustered cells were visualized using UMAP. Before anchoring and integration, macrophage identities from both datasets were renamed to match macrophage identities from the current study. Briefly, cells from WT (monocyte_patrolling, monocyte_inflammatory, monocyte_mixed, M2 macrophage_*Cx3cr1*^lo^, M2 macrophage_*Cx3cr*^hi^, and dendritic cells) and mdx (*Lyz2*, *Ctss*, *Cd68*, *Fcgr2b*, *Cd14*, and *Adgre1*-expressing cells) were subclustered and subjected to reference-based mapping using the macrophage identities described in this study (i.e., reference cells – macrophage 0 to 5) (*81*). Following a similar approach, WT FAP_adipogenic, FAP_proremodeling, FAP_stem, and tenocytes (*37*) were used as reference to assign identities to *Pdgfra*^+^ FAPs and tenocytes in the mdx dataset. All other previously assigned cell identities described in Oprescu *et al.* (*37*) were kept in the final clustering except for capillary, mixed, and vein endothelial cells, which were collapsed into “endothelial cells”; T and natural killer (NK) cells, which were separated into T and NKT cells; and cycling cells, which were identified by expression of *Mki67*.

#### Reference-based mapping of B6AJ and limb girdle macrophages

We performed reference-based mapping, anchoring, and integration of muscle macrophage scRNA datasets prepared from two healthy and two dystrophic mice using Seurat (version 3.2.2, R Studio version 3.6.1) (*81*). Datasets were prepared from 8-month-old B6A/J (LGMD2B, *n* = 2) and B6 (WT, *n* = 1). The annotated macrophage dataset in Fig. 1 was used as the reference. WT B10, mdx, WT B6, and B6A/J objects were merged, SCTransform-normalized, anchored, and integrated as described above. Clustered cells were visualized using two-dimensional UMAP.

#### Modeling cell-cell communication networks

Intra- and intercellular communication networks were modeled based on the basis of abundance of known ligand-receptor (L-R) transcript pairs with CellChat (version 1.1.3) (*36*). To identify conserved and perturbed FAP-macrophage communication networks in WT and mdx muscles, we lifted cells from a WT-mdx integrated object. We performed joint manifold and classification learning analyses as described in CellChat.

### Bulk RNAseq and analysis

Total RNA was monitored for quality control using the Agilent Bioanalyzer Pico RNA chip (Agilent Technologies, Santa Clara, CA) and NanoDrop (Thermo Fisher Scientific, Waltham MA) absorbance ratios for 260/280 and 260/230 nm. Library construction was performed according to the SMARTer Stranded Total RNA-Seq Kit v2-Pico Input Mammalian (Takara Bio, Mountain View, CA). The input quantity for total RNA was 2 ng. The total RNA was fragmented for 3 min at 94°C. SMART (Switching Mechanism At 5′ end of RNA Template) cDNA synthesis technology was used to synthesize cDNA from the fragmented total RNA. Illumina adapters were ligated to the ends and enriched by 5 cycles of PCR. R-probes v2 (mammalian specific) are then hybridized to the cDNA that contains ribosomal RNA and human mitochondrial ribosomal RNA sequences. The R-probe v2 hybridized cDNAs are then cut by ZapR v2. The leftover library fragments are further enriched by 14 cycles of PCR. The final libraries are purified via AMPure XP beads. The resulting libraries were validated by qPCR (Kapa library quantification kit, Kapa Biosystems, Wilmington MA) and sized by an Agilent Bioanalyzer DNA high-sensitivity chip. The concentrations for the libraries were normalized and then multiplexed together. The multiplexed libraries were sequenced using paired-end 100 chemistry on the NovaSeq 6000 (Illumina, San Diego, CA).

Public data for microglia (*33*) (GSE132877) and bone-derived macrophage (*34*) (provided by authors) RNAseq were processed from original fastq read files. All reads were mapped to the mouse genome (mm10) (*82*) with TopHat (*83*) (version 2.0.14) with reference GENCODE transcript annotation (*84*) (M9). Overlapping reads were counted and summarized by gene using HTSeq (*85*) (1.99.2). The R package DESeq2 Field (*85*) (version 1.28.1) was used to determine DEGs between datasets. From DESeq2 output, DEGs were classified for twofold changes up or down with a false discovery rate < 0.01. Gene sets from the Molecular Signature Database (*86*) (mSigDB) were downloaded from the Gene Set Enrichment Analysis webpage (http://software.broadinstitute.org/gsea) to determine gene set enrichment. Plots were generated in R using Venn diagram (version 1.6.20), ggrepel (version 0.9.0), ComplexHeatmap (version 2.4.3), plyr (version 1.8.6), and ggplot2 (version 3.3.3).

### Spatial transcriptomics

The 10x Genomics Visium Spatial Gene Expression platform was used for spatial transcriptomics analysis of muscle from dystrophic mice (D2-mdx, stock no. 013141 from The Jackson Laboratory) according to the manufacturer’s guidelines. Briefly, 6-week-old male mice were euthanized via cervical dislocation under isoflurane anesthesia. The gastrocnemius/plantaris muscle complex was immediately dissected and frozen in optimal cutting temperature (OCT) embedding media in liquid nitrogen–cooled isopentane. Muscle tissue was cryosectioned at −20°C at 10-μm thickness onto Visium Spatial Gene Expression slides (10x Genomics) and stored at −80°C until processing. Sections were fixed in prechilled methanol for 30 min. Hematoxylin and eosin (H&E) staining was performed per the published protocol from 10x Genomics, with imaging performed using a Zeiss Axio Observer microscope. H&E images were stitched and processed using Zen 2.0 software. Following imaging, tissues were permeabilized for 12 min, which was predetermined as the optimal time for 10-μm mouse muscle sections using the 10x Genomics Visium Tissue Optimization Kit. Spatially tagged cDNA libraries were built using the 10x Genomics Visium Spatial Gene Expression Library Construction Kit. Sequencing was performed on an Illumina NextSeq 500/550 using 150-cycle high output kits (read 1 = 28, read 2 = 120, index 1 = 10, and index 2 = 10). Alignment to the mouse reference genome mm10 (Ensembl 93) was done using the Space Ranger 1.0.0 pipeline to derive a feature spot-barcode expression matrix (10x Genomics). Alignment of H&E images was done using Loupe Browser.

### Adoptive transfer

Monocytes were isolated from bone marrow using the EasySep Mouse Monocyte Enrichment kit (STEMCELL Technologies). Briefly, femurs and tibias were harvested and flushed through with cold phosphate-buffered saline (PBS). Bone marrow cell suspensions were passed through a 70-μm cell strainer to obtain a single-cell suspension. Red blood cells were removed using the red blood cell lysis buffer according to the manufacturer’s instructions (Sigma-Aldrich). The bone marrow cell suspension was treated with the EasySep reagents, and monocytes were isolated by depletion using an EasyPlate magnet (STEMCELL Technologies). Muscle immune cells were isolated from hindlimb muscles as described previously and enriched using the EasySep Release Mouse Allophycocyanin (APC) selection kit (STEMCELL Technologies). Briefly, the muscle single-cell suspension was resuspended in 0.25 ml of recommended medium and incubated with the Folr2-APC antibody (2 μg/ml) for 5 min on ice. Following incubation with 25 μl of APC selection cocktail for 5 min at 4°C, APC^+^ cell selection is obtained by incubating with RapidSpheres magnetic beads on ice for 3 min. Folr2^+^ macrophages bound to the magnetic beads were eluted after adding the ice-cold release buffer on ice for 3 min, counted, and saved for downstream experiments. Isolated monocytes (20,000 cells/μl in PBS) or Folr2^+^ macrophages (15,000 cells/μl in PBS) were injected in 10 μl intramuscularly. Briefly, mdx mice were anesthetized with isoflurane while placed over a heating pad to maintain thermoregulation. Anesthetized mice were sterilized with 70% ethanol, and 10 μl of cell suspension was injected into the quadriceps using a Hamilton syringe. When appropriate, the other quadriceps was used as a control where no cells were injected into the muscle. Flow analysis of the quadriceps of mdx mice was performed 2 and 7 days following the adoptive transfer.

### Statistical analyses

Data were expressed as mean ± SEM. Statistical analyses were performed using GraphPad Prism version 9.2. Statistical comparisons between the two groups were performed using an unpaired *t* test with Welch’s correction. One-way or two-way analysis of variance (ANOVA) with a post hoc Bonferroni test, Kruskal-Wallis multiple comparisons test, or Sidak’s multiple comparisons tests was conducted when comparing multiple groups. *P* values δ of 0.05 were considered significant.
