# Supplementary material for: Single-cell and spatial transcriptomics identify a macrophage population associated with skeletal muscle fibrosis
Source: Sci Adv. 2023 Jul 7;9(27):eadd9984. doi: 10.1126/sciadv.add9984 (PMC10328414; doi:10.1126/sciadv.add9984)
Supplement: Supplementary file 1 — Supplementary Materials and Methods Figs. S1 to S13 Legend for table S1 Table S2 [file sciadv.add9984_sm.pdf]

Science Advances
AAAS

Supplementary Materials for

Single-cell and spatial transcriptomics identify a macrophage population associated with skeletal muscle fibrosis

Gerald Coulis *et al.*

Corresponding author: S. Armando Villalta, villalts@uci.edu

Sci. Adv. **9**, eadd9984 (2023)
DOI: 10.1126/sciadv.add9984

The PDF file includes:

Supplementary Materials and Methods
Figs. S1 to S13
Legend for table S1
Table S2

Other Supplementary Materials for this manusript includes the following:

Table S1

SUPPLEMENTARY MATERIALS and METHODS

Tissue cell isolation

Skeletal muscle

Single-cell isolation from hind-limb muscles was performed as previously described (*87*). Briefly, following whole body perfusion with phosphate-buffered saline (PBS), hind-limb muscles were harvested from the mice while removing the popliteal lymph node to exclude any potential contamination of non-muscle-residing leukocytes in our preparations. Wild type and mdx hind limb musculature were processed and analyzed individually unless specified otherwise. Minced hind limb muscles were subjected to 2, 30-minute rounds of enzymatic digestion with 0.2 mg/ml collagenase P (Roche) and 20 μg/ml of DNAse (Roche) in serum-free Dulbecco's Modified Eagle Medium (DMEM). Following digestion, single-cell preparations were sequentially filtered through a 70 and 40 μm cell strainer. The filtrate was suspended over 5 ml of Lymphoprep (Stem Cell Technologies) and centrifuged at ~315 x g for 30 minutes at room temperature. Cells at the interface were harvested, counted, and analyzed or sorted by flow cytometry.

Heart and quadriceps

Immune cells from the heart or quadriceps were isolated using a modification of the above procedure. The heart or quadriceps were minced, then digested twice for 20 minutes in a 0.2 mg/ml collagenase P solution supplemented with 20 μg/ml of DNase. The muscle suspension was then sequentially filtered through 70 and 40 μm cell strainers. Following a final suspension using a 35-μm cell strainer, immune cells were counted and stained for flow cytometry analysis.

Liver

Immune cells from the liver were isolated as described (*88*) with some modifications. Briefly, following whole body perfusion with PBS, liver lobes were minced and digested in 0.2

mg/ml collagenase P (Roche) and 20 µg/ml of DNAse (Roche) in serum-free DMEM at 37 °C with two rounds of 20-minute enzymatic digestion. Red blood cells (RBC) were lysed using 1X RBC lysis buffer. Cell pellets were washed with 1X PBS, resuspended and filtered through 70 and 40 µm cell strainers. Cells were counted prior to cell surface staining.

Bone marrow

Primary mouse bone marrow cells were isolated by flushing the tibiae and femurs with PBS. The resulting cell suspension was gently disaggregated and passed through a 70 µm cell strainer to produce a single cell suspension. Red blood cells were lysed by incubating for 5 minutes with ACK lysis buffer. Bone marrow cells were counted prior to cell surface staining.

Mouse PBMCs

Mouse blood was collected by cardiac puncture with a heparin-coated syringe. Blood was transferred to an Eppendorf tube containing 10 µl of heparin (1000 USP/ml). The blood was diluted with an equal volume of PBS and gently layered on top of the density gradient medium, Lymphoprep (Stem Cell Technologies). Cells were harvested from the interface following a centrifugation at 315 x g for 20 minutes. PBMCs were then washed twice with PBS prior to downstream applications.

Brain

Central nervous system (CNS) macrophages were sorted from single-cell suspensions prepared from the brain and spinal cord as previously described (*89*). Briefly, brains and spinal cords were dissected from PBS-perfused mice and mechanically dissociated using a Dounce grinder (# K8853000007, ThermoFisher) to obtain tissue homogenates. CNS tissue homogenates were further digested with Collagenase IV (Worthington-Biochemical) (1 mg/ml) and DNase I (Sigma Aldrich) (200 Units/ml) in 5 ml of RPMI 1640 media for 45 minutes at 37 °C. To separate

myelin debris from cells, digested tissue was passed through a 100 µm cell strainer, followed by density centrifugation with 23% Percoll (GE Life Sciences Cat# 17089101). Gradients were centrifuged at 400g without brakes for 25 minutes at 4 °C. The myelin layer at the interface was removed and the single cells in the pellet were collected. Additional RBC lysis was performed using ACK buffer (# A1049201, ThermoFisher) for 60 seconds on ice when necessary. The single-cell suspension was then passed through a 70 µm cell strainer, washed twice with PBS for subsequent FACS and RNAseq analysis.

Skin

Single-cell suspensions were prepared by mincing whole skin samples from shaved mice, followed by digestion with 10 ml of 0.25% collagenase (Sigma, C9091) and 0.1 mg/mL DNase (Sigma, DN25) in 0.01 M HEPES (ThermoFisher, BP310) and 0.001 M sodium pyruvate (ThermoFisher, BP356). Skin was digested at 37 °C for 1 hour with agitation, and then filtered through 70 µm and 40 µm cell strainers, spun down, and resuspended in 2% fetal bovine serum (FBS) in PBS (Alphabioregen, Alpha FBS). Isolated cells were counted prior to cell staining.

Lung

Lung single-cell suspensions were prepared using mechanical and enzymatic digestion. The trachea was exposed and 1 ml of digestion media composed of RPMI containing 0.1 mg/ml collagenase P and 0.02 mg/ml DNase was injected into the trachea to inflate the lungs. The lungs were then dissected, minced, and digested in 3 ml of digestion media for 45 minutes while rocking at 37 °C. Following digestion, suspensions were diluted with HBSS and filtered through a 70µm cell strainer and pelleted using centrifugation. Red blood cells were lysed by incubating cells with ACK lysis buffer (Life Technologies) for 5 minutes. Single-cell suspensions were then analyzed using flow cytometry.

RNAscope

6-wk-old male mice were euthanized via cervical dislocation under isoflurane anesthesia, and the gastrocnemius/plantaris muscle complex was immediately dissected and frozen in OCT embedding media in liquid nitrogen-cooled isopentane. Muscle tissue was cryosectioned at -20 °C at 10 μm thickness onto Superfrost Plus micro slides (VWR) and stored at -80 °C until processing. Slides were prepared for the RNAscope® assay according to the Advanced Cell Diagnostics (ACD) protocol for fresh frozen tissue (Document number 320513, ACD). RNAscope® was performed according to the manufacturer's protocol, RNAscope® Multiplex Fluorescent Assay V2 (Document Number 320293-USM, ACD), using RNAscope® Probe - Mm-Spp1-C2 (ACD, catalog #435191-C2) with probe diluent (ACD, catalog #300041) and Amp4 Alt C-FL (Atto 550). Prior to counterstaining, the section was incubated in blocking solution (20% goat serum, 0.3% Triton X-100 in 1X PBS) with 1:100 Laminin (Sigma, L9393) for 30 minutes. Slides were washed with PBS two times for 1 minute and incubated in a filtered blocking solution with 1:250 Alexa Fluor 488 (ThermoFisher, 32731). Slides were washed once with PBS for 1 minute. Counterstaining was performed as directed by the manufacturer's protocol, followed by mounting with Fluoromount G (ThermoFisher, 00-4958-02). Sections were imaged using a Zeiss Axioscan microscope and processed using HALO software (Indica Labs).

Barium Chloride injury

Prior to muscle injury, 6-7-wk-old mice were anesthetized using a mixture of 2-3% L/minutesof isoflurane and oxygen. Barium chloride-induced acute muscle injury was carried out by 4, 20 μl intramuscular injections of barium chloride dissolved in saline solution (1.2% w/v), applied along the length of the quadriceps to maximize the distribution of barium chloride. The

contralateral quadriceps served as uninjured control. Following injury, animals were euthanized at different time points, and quadriceps were harvested for flow cytometry or histological analysis.

Histological analyses

Picrosirius Red

Muscle cryosections were fixed overnight in Bouin's fluid at room temperature and cleared in xylene prior to hydration with decreasing ethanol concentrations. Sections were stained for 15 minutes at room temperature under agitation in 0.02% picrosirius red solution (Abcam). Following washes in acetic acid, the slides were dehydrated and mounted with non-aqueous mounting media. The collagen content labeled by the picrosirius red staining is examined using a Cytation 5 brightfield microscope.

Hematoxylin and Eosin

Hematoxylin and eosin stain was performed on fresh frozen muscle tissues. Quadriceps muscle cryosections (8 μm) were stained with Harris hematoxylin and then counterstained with eosin. Sections were then examined using a Cytation 5 brightfield microscope.

Immunofluorescence

Serial sections were fixed with 2% paraformaldehyde for 5 minutes. Slides were washed with PBS three times for 5 minutes and incubated in blocking solution for 1 hour. Slides were then washed with PBS three times for 5 minutes and incubated in primary antibody solution for 3 hr or overnight in a humidifying chamber. Primary antibodies were as follows: PDGFRα (R&D Systems, AF1062, 1:200), galectin-3 (Biolegend, 25401, 1:300), Folr2 (Biolegend, 153302, 1:200), laminin (Sigma-Aldrich, L9393 or Abcam, ab11576 at 1:500), collagen I (Abcam, ab270993, 1:200), or F4/80 (Abcam, ab6640, 1:50). Slides were then washed three times for 5 minutes with PBS and then incubated with secondary antibodies (diluted 1:250 in filtered blocking

solution). Secondary antibodies were as follows: rabbit Alexa Fluor 546 (ThermoFisher, 11035, 1:500), rat Alexa Fluor 488 (ThermoFisher, 11006, 1:200), rat Alexa Fluor 647 (ThermoFisher, 21247, 1:200), or biotinylated anti-rat (ThermoFisher, A18749, 1:200) coupled with streptavidin-conjugated Alexa Fluor 488 (ThermoFisher, S11223, 1:500). Slides were washed once with PBS for 5 minutes and then incubated in DAPI (ThermoFisher) for 5 minutes. After the DAPI stain, three additional, 5-minute PBS washes were performed and slides mounted with Fluoromount G (ThermoFisher, 00-4958-02). Sections were imaged using a Zeiss Axioscan microscope and processed using HALO software.

RNA isolation and qPCR analysis

Liquid nitrogen-frozen muscle samples were homogenized, and RNA was extracted using TRIsure (Bioline) and the Quick-RNA Miniprep kit (Zymo Research) per manufacturer instructions. Cell populations were sorted directly into lysis buffer, and RNA was isolated using the Quick-RNA Microprep kit (Zymo Research). Complementary DNA (cDNA) was synthesized from 150 ng (sorted cells) or 1000 ng (whole muscle) of DNase-treated RNA using the SensiFAST cDNA synthesis kit (Bioline). Gene expression was quantified using TaqMan expression assay probes (Life Technologies) and 2X SensiFAST probe No-ROX mix (Bioline). All gene expression was normalized to 18S unless otherwise noted.

Flow cytometry

To discriminate between live and dead cells, cells were stained with 100 µl of Zombie NIR viability dye (1:1000 in 1X PBS) for 15 minutes on ice while protected from light. Fc-receptor blocking of muscle single-cell suspensions was performed by incubating cells with an anti-CD16/32 antibody (clone 2.4G2) prior to staining. Single-cell suspensions were stained with a panel of antibodies against several cell surface antigens (see **table S2**). Analysis was performed

on a BD FACSAria Fusion flow cytometer with FACSDiva software (BD Bioscience). Post-acquisition analysis was performed with Flowjo software version 10.8.

SUPPLEMENTARY FIGURES

Supplementary Figure 1

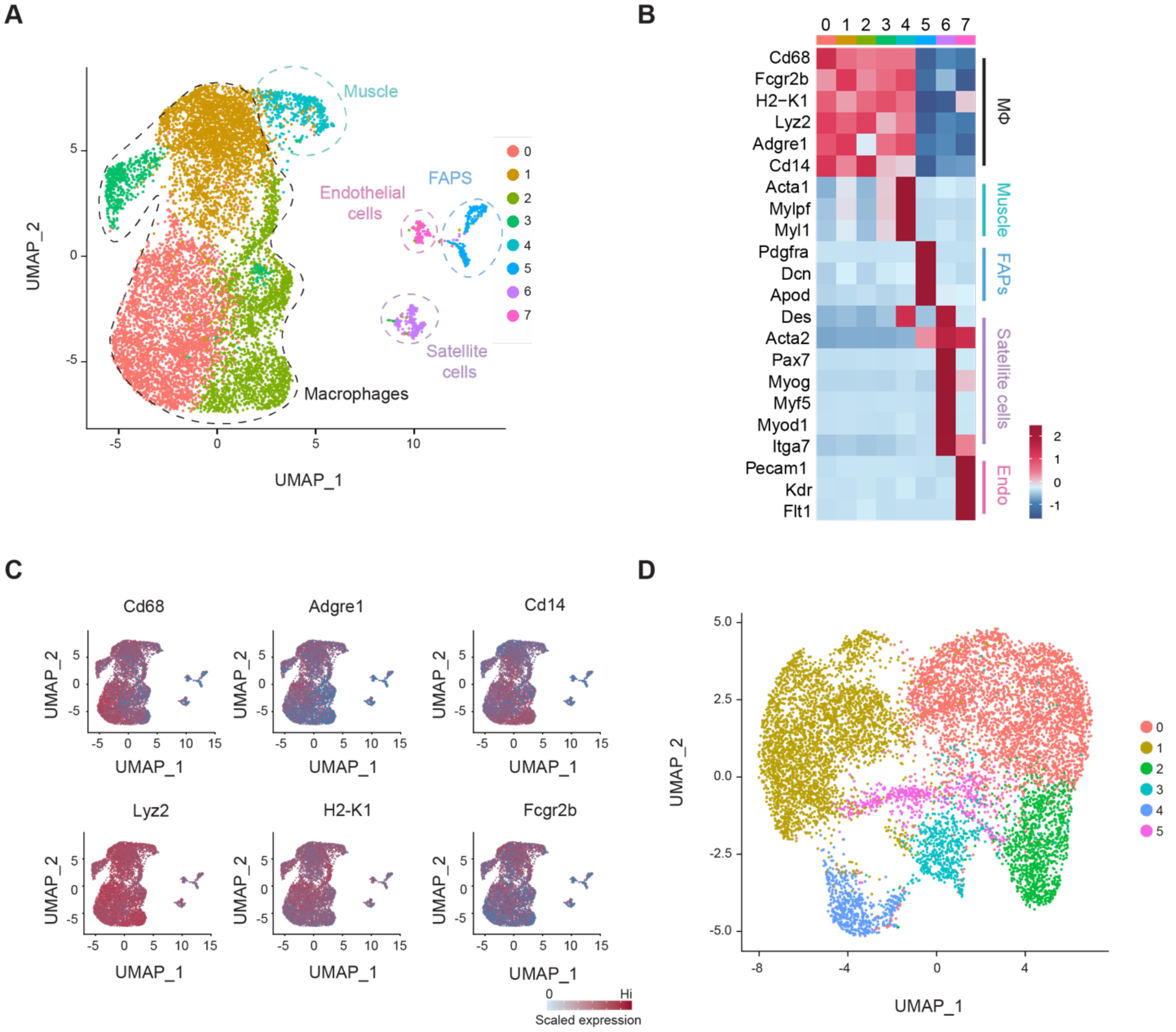

Fig. S1. Unsupervised learning and clustering of muscle macrophage scRNAseq data. (**A**) Dimensionality reduction via UMAP showing all cells in the WT and mdx muscle macrophage datasets. (**B** and **C**) heatmap showing cell-specific marker genes (B) and feature plots showing the expression of macrophage marker genes (C). (**D**) UMAP showing sub-clustering analysis of muscle macrophages shown in A. Six novel populations were identified.

Supplementary Figure 2

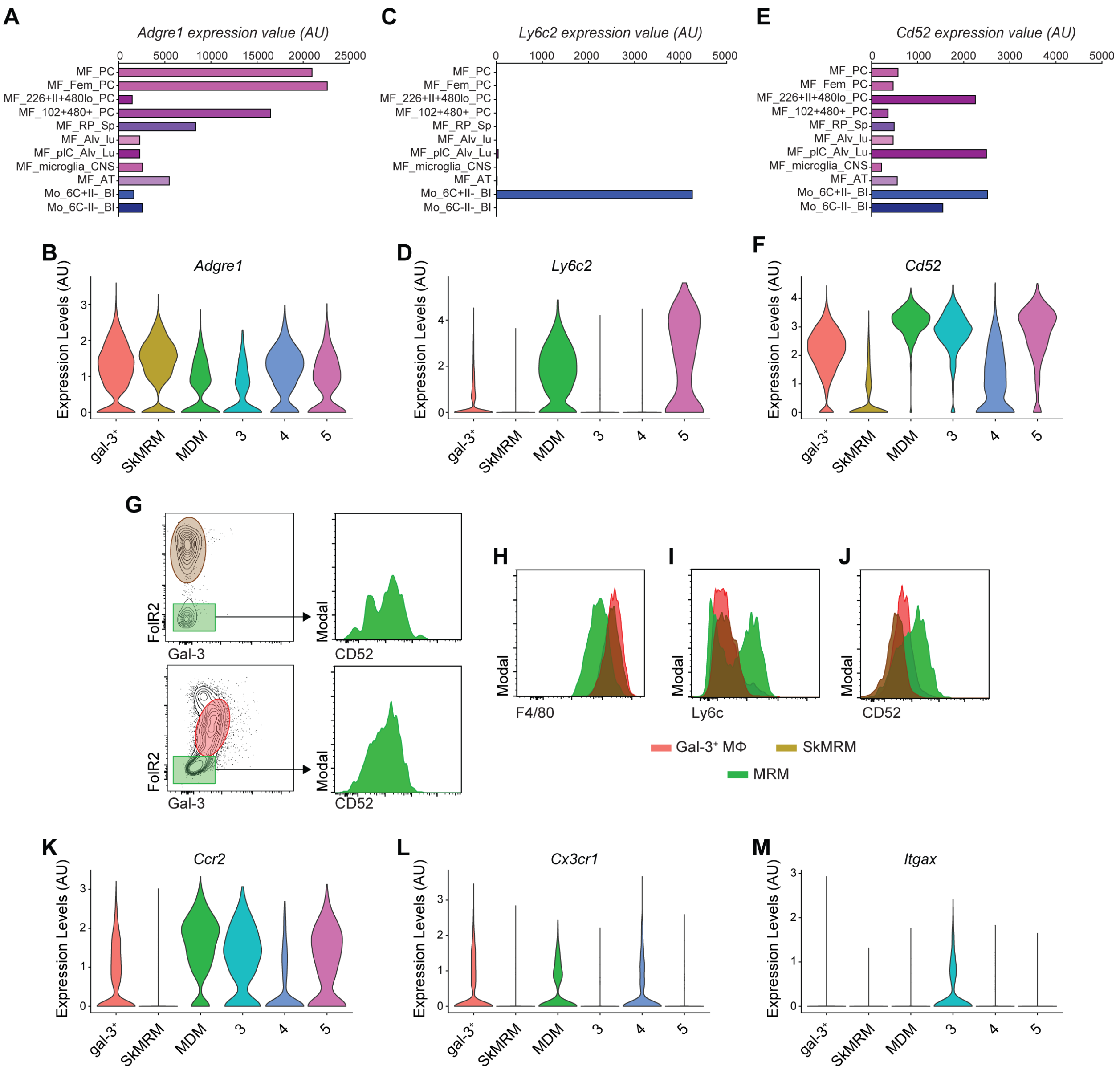

Fig. S2. Cluster 2 macrophages in dystrophic muscle resemble monocytes. (**A-F**) Expression of key monocytes markers. The expression of levels of *Adgre1* (F4/80) (A), *Ly6c* (C) and *CD52* (E) in monocyte and macrophages populations were assessed through the ImmGen database (www.immgen.org). Violin plots showing expression of *Adgre1* (B), *Ly6c* (D) and *CD52* (F) in the scRNAseq analysis (Fig. 1). MF.Fem.PC= Female Peritoneal Macrophages obtained by sorting F4/80^{+}ICAM^{+}CD5^{-}CD19^{-}CD43^{-} cells; MF.226+II+480lo.PC= Peritoneal Small Macrophages obtained by sorting CD115^{+}CD11b^{+}F4/80^{lo}CD102^{lo}MHCII^{+}CD226^{+}; MF.102+480+.PC= Peritoneal Large Macrophages obtained by sorting CD115^{+}CD11b^{+}F4/80^{+}CD102^{+}MHCII^{lo} CD226^{-} ; MF.RP.Sp= Red pulp Macrophages from spleen sorted on CD11b^{lo}F4/80^{+}Mertk^{+}CD64^{+}; MF.Alv.Lu= Lung Alveolar Macrophages sorted on CD45^{+}CD11c^{+}SiglecF^{+}; MF.pIC.Alv.Lu=

Lung Alveolar Macrophages stimulated with Polyinosinic-polycytidylic acid [poly(I:C)]; MF.microglia.CNS= Brain Microglia Macrophages; MF.AT= Inguinal and Perigonadal Adipose Tissue Macrophages; Mo.6C+II-.Bl= Ly6C^{hi} blood monocytes sorted on B220^{-}CD3^{-}Ly6G^{-}CD45^{+}CD115^{+}CD11b^{+}Ly6C^{+}; Mo.6C-II-.Bl= Ly6C^{lo} blood monocytes sorted on B220^{-}CD3^{-}Ly6G^{-}CD45^{+}CD115^{+}CD11b^{+}Ly6C^{-}. (**G-J**) Representative gating strategy for interrogating the expression of F4/80 (H), Ly6c (I) and CD52 (J) in Gal-3^{+} Mϕ, SkMRMs and MDMs. (**K-M**) Violin plots showing the expression levels of monocyte-related genes, *Ccr2* (K), *Cx3cr1* (L), and the dendritic cell marker, *Itgax* (CD11c) (M).

Supplementary Figure 3

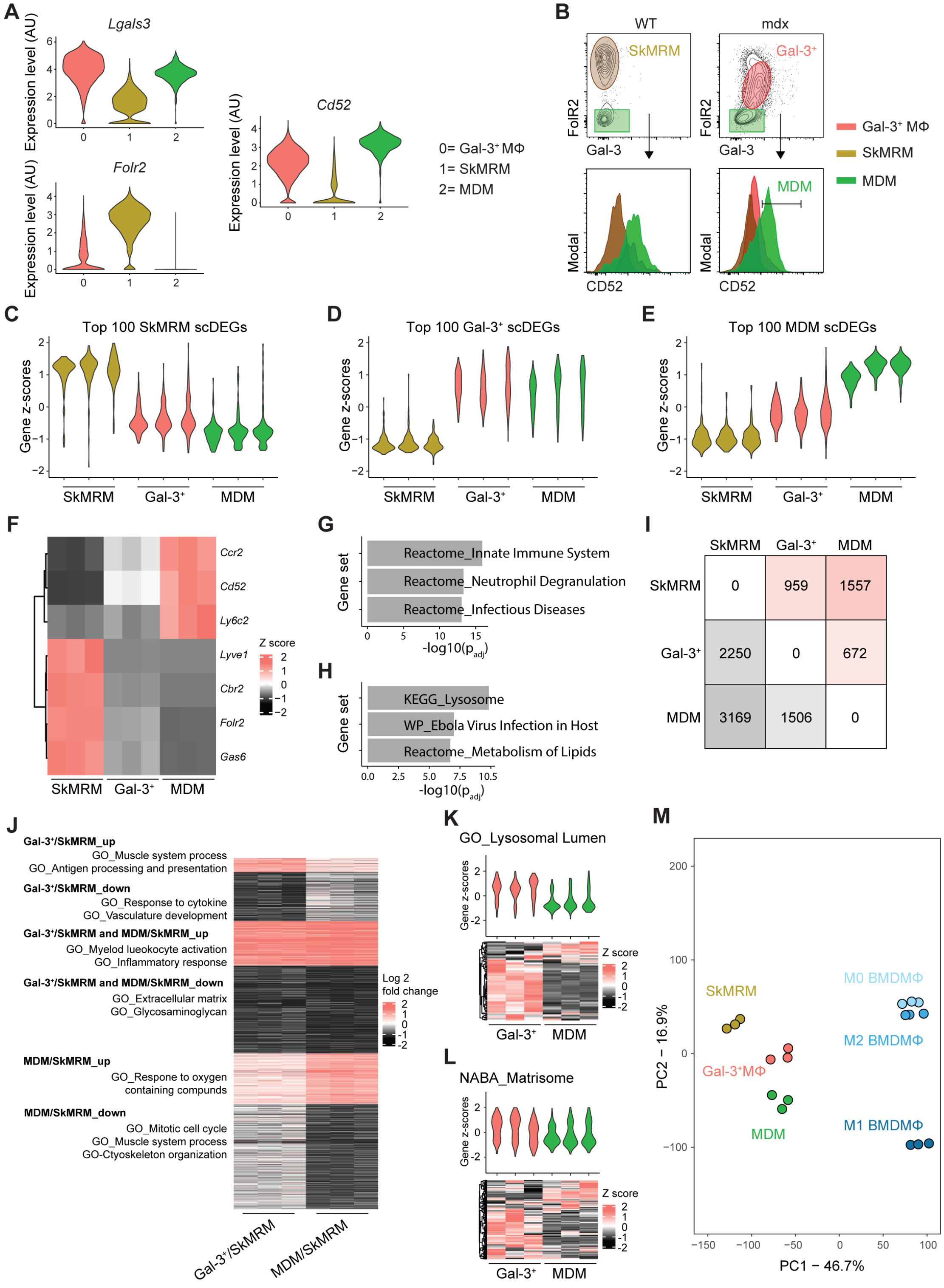

Fig. S3. Characterization of SkMRM, gal-3^{+} macrophages and MDMs.
(A) Violin plots of clusters 0 (Gal-3^{+} macrophages), 1 (SkMRM) and 2 (MDM) marker genes. **(B)** Representative flow cytometry plots showing the gating strategy for Gal-3^{+} Mϕ, SkMRM and

MDM. (**C-E**) Violin plots showing the z-scores for the top 100 scDEGs from SkMRMs (C), Gal-3^+ Mϕ (D) and MDMs (E) in the transcriptomes of sorted populations. (**F**) Heatmap showing the expression of representative marker genes from the scRNAseq analysis in the transcriptomes of sorted macrophage populations. (**G and H**) Pathway analysis showing the top 3 gene sets enriched in principal component 1 (G) and principal component 2 (H) in the PCA shown in figure 2D. (**I**) A matrix showing the number of genes upregulated (pink) or downregulated (grey) in SkMRM, gal-3^+ Mϕ or MDMs. $FDR < 0.01$, $FC > 2$. (**J**) Heatmap with selected GO pathways enriched in gal-3^+ Mϕ, MDMs or both. (**K and L**) Enrichment of genes associated with the lysosomal lumen (K) and the matrisome (L) in gal-3^+ Mϕ or MDMs, visualized by heatmap and violin plot. (**M**) Principal component analysis applied to muscle and bone marrow-derived macrophages (BMDMϕ).

Supplementary Figure 4

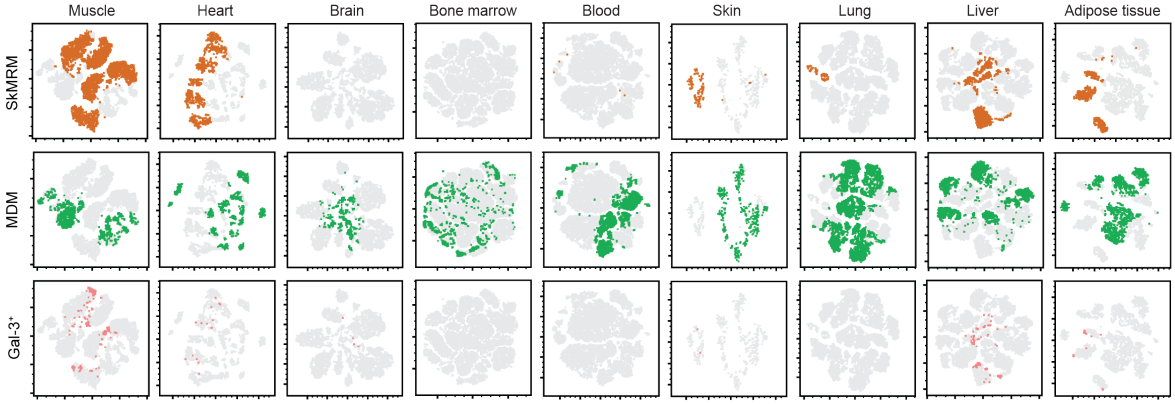

Fig. S4. Prevalence of SkMRM, MDM and gal-3^{+} macrophages in healthy tissues. Concatenated TSNE flow plots of live CD11b^{+}F4/80^{+}Siglec-F^{-} cells from 4-wk-old WT mice. n= 5 per organ or tissue. Grey shows CD11b^{+}F4/80^{+}Siglec-F^{-} cells. Orange indicates the proportion of CD11b^{+}F4/80^{+}Siglec-F^{-} cells that are Gal-3^{lo}Folr2^{hi}, green indicates the proportion that are CD52^{+}, and pink indicates the proportion that are Gal-3^{hi}Folr2^{lo}.

Supplementary Figure 5

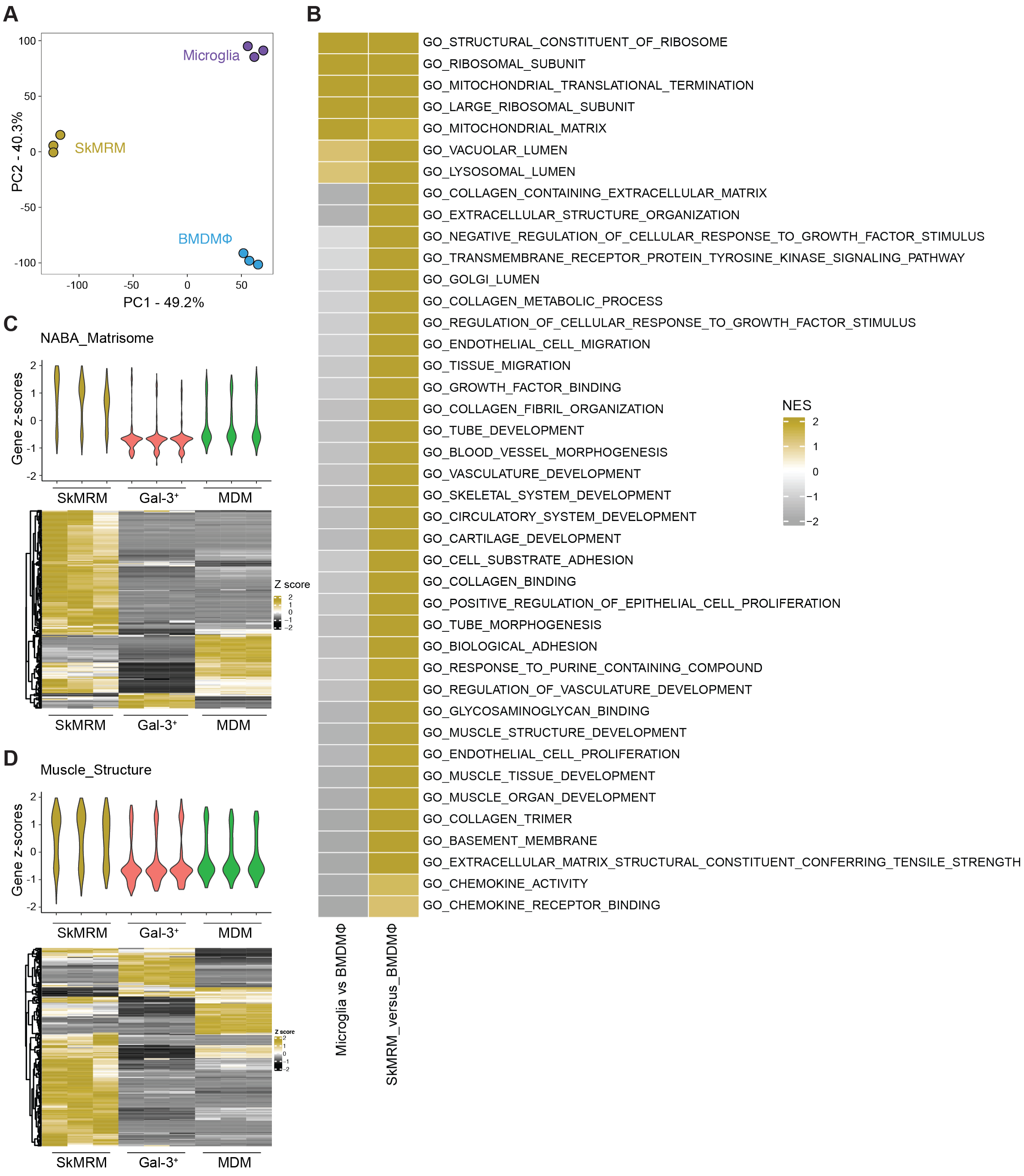

Fig. S5. Skeletal muscle-resident macrophages express a transcriptome associated with muscle homeostasis and function. (**A**) Principal component analysis of SkMRM, microglia and BMDMϕ. (**B**) Heatmap summarizing GSEA analysis with Gene Ontology (GO) pathways that indicate enrichment of muscle- and ECM-related pathways in SkMRMs. NES= Normalized Enrichment Score. (**C and D**) Violin plots of gene z-scores and corresponding heatmaps showing the relative expression of genes associated with the matrisome (C) and muscle structure (D) in SkMRMs.

Supplementary Figure 6

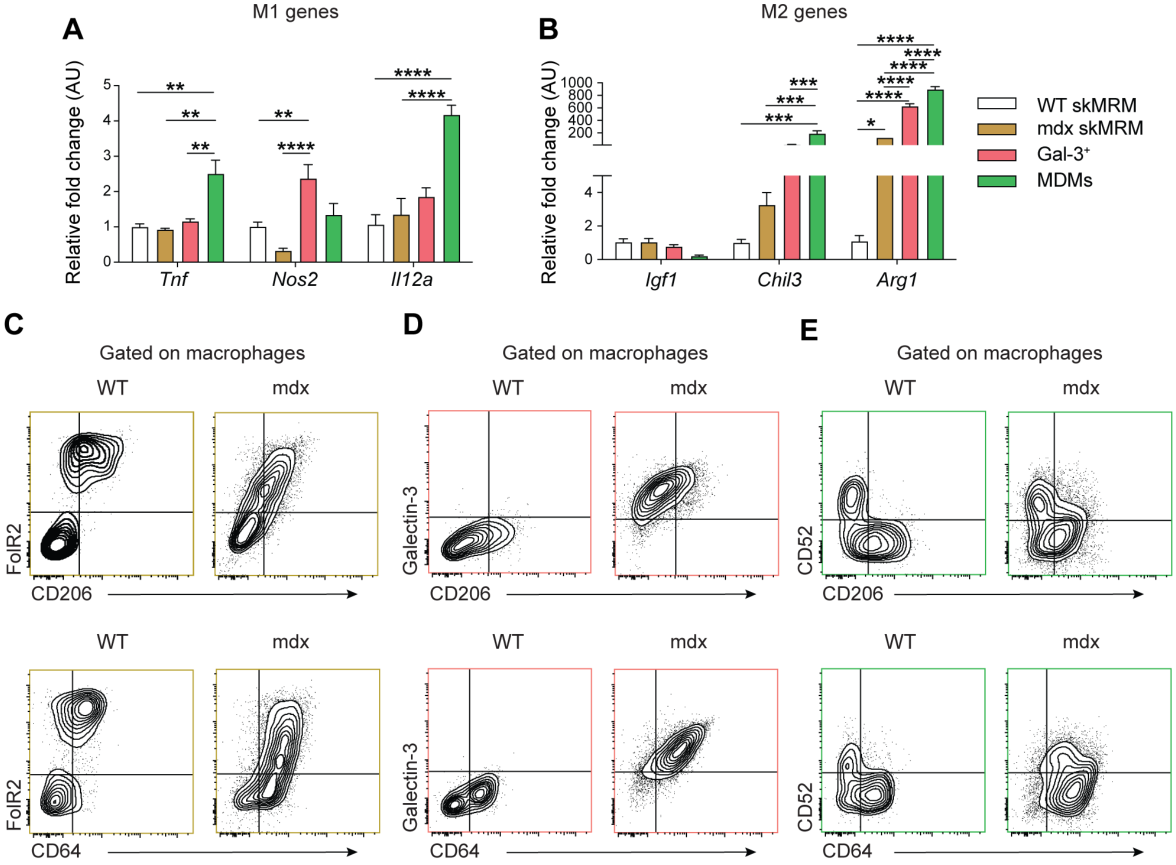

Fig. S6. Novel skeletal muscle macrophage populations heterogeneously express qualities of M1 and M2 macrophages. (**A** and **B**) Expression of key M1 (A) and M2 markers genes in sorted WT SkMRM, mdx SkMRM, Gal-3^{+} Mϕ and MDMs from mdx mice. n= 3 sorted macrophages for each subpopulation. A two-way ANOVA with a Tukey multiple comparison test was performed. (**C-E**) Representative contour plots showing the differential expression of CD206 (M2 marker) and CD64 (M1 marker) in SkMRM, Gal-3^{+} Mϕ and MDMs from 4-wk-old WT and mdx mice. n= 5. *p<0.05, **p<0.01, ***p<0.001, ****p<0.0001 using or 2-way ANOVA with Tukey's multiple comparison test (A and B).

Supplementary Figure 7

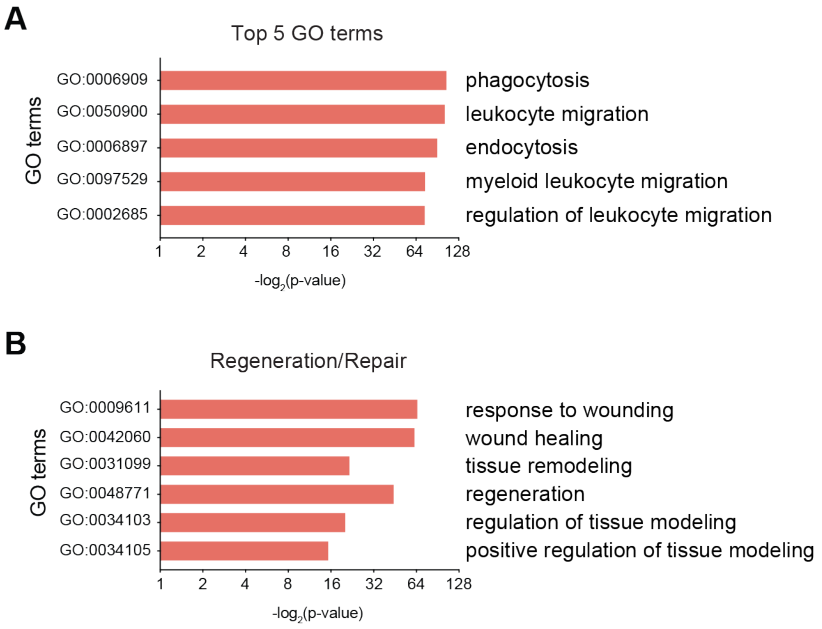

Fig. S7. Top 5 and regeneration/repair-associated GO terms. (**A** and **B**) Gene ontology analysis performed on the differentially expressed genes between *Lgals3*^{lo} and *Lgals3*^{hi} spots from the spatial transcriptomics. The top 5 GO terms (A) and the regeneration and repair terms (B).

Supplementary Figure 8

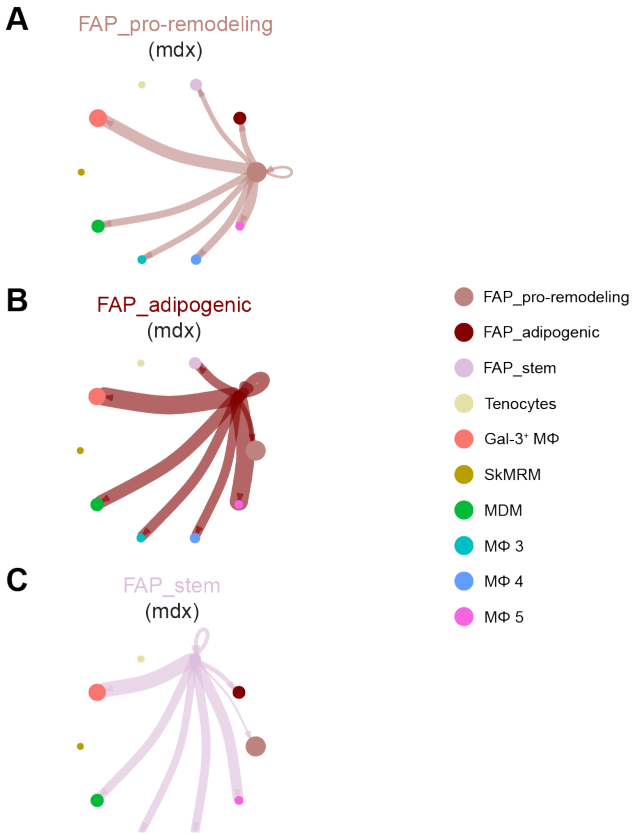

Fig. S8. Visualization and analysis of cell-cell communication between FAP and macrophage populations using CellChat. (**A-C**) Circle plots placing pro-remodeling (A), adipogenic (B) and stem-like FAPs (C) as the central nodes of analysis in the mdx dataset.

Supplementary Figure 9

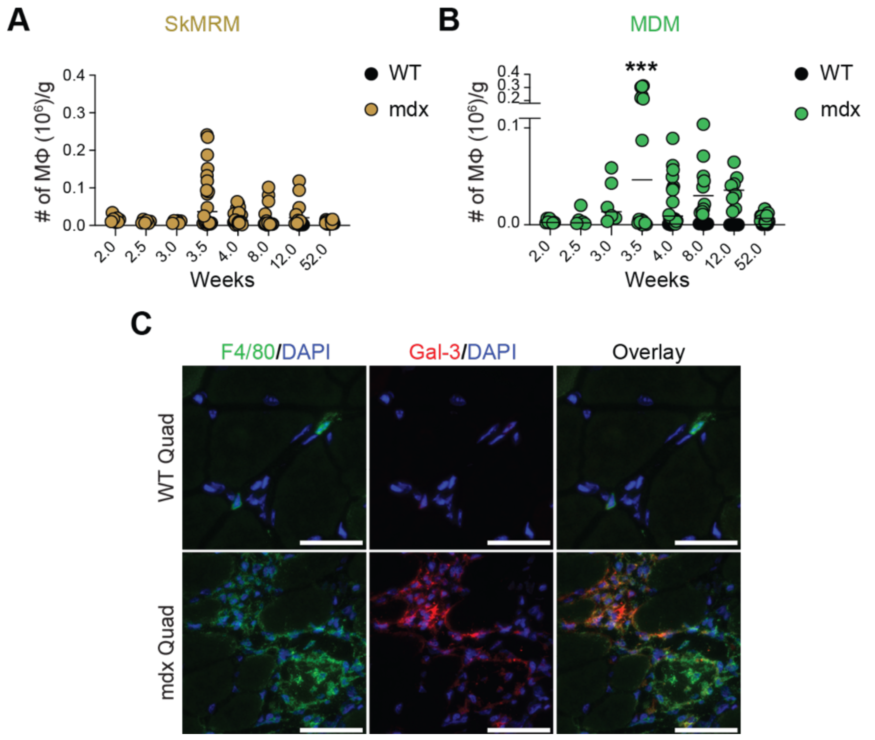

Fig. S9. Regulation and localization of muscle macrophage populations in muscular dystrophy. (**A** and **B**) Enumeration of SkMRMs (A) and MDMs (B) during muscular dystrophy. n= 7-9 for each time point. (**C**) Immunofluorescence staining of macrophages in dystrophic muscle. Immunofluorescence localization of Gal-3^{+} Mϕ using a galectin-3-specific antibody (red). F4/80 was used a pan macrophage marker (green). DAPI was used to counterstain nuclei (blue). Scale bar, 50 µm. *$p<0.05$, **$p<0.01$, ***$p<0.001$, ****$p<0.0001$ using a 2-way ANOVA with Sidak's multiple comparison test.

Supplementary Figure 10

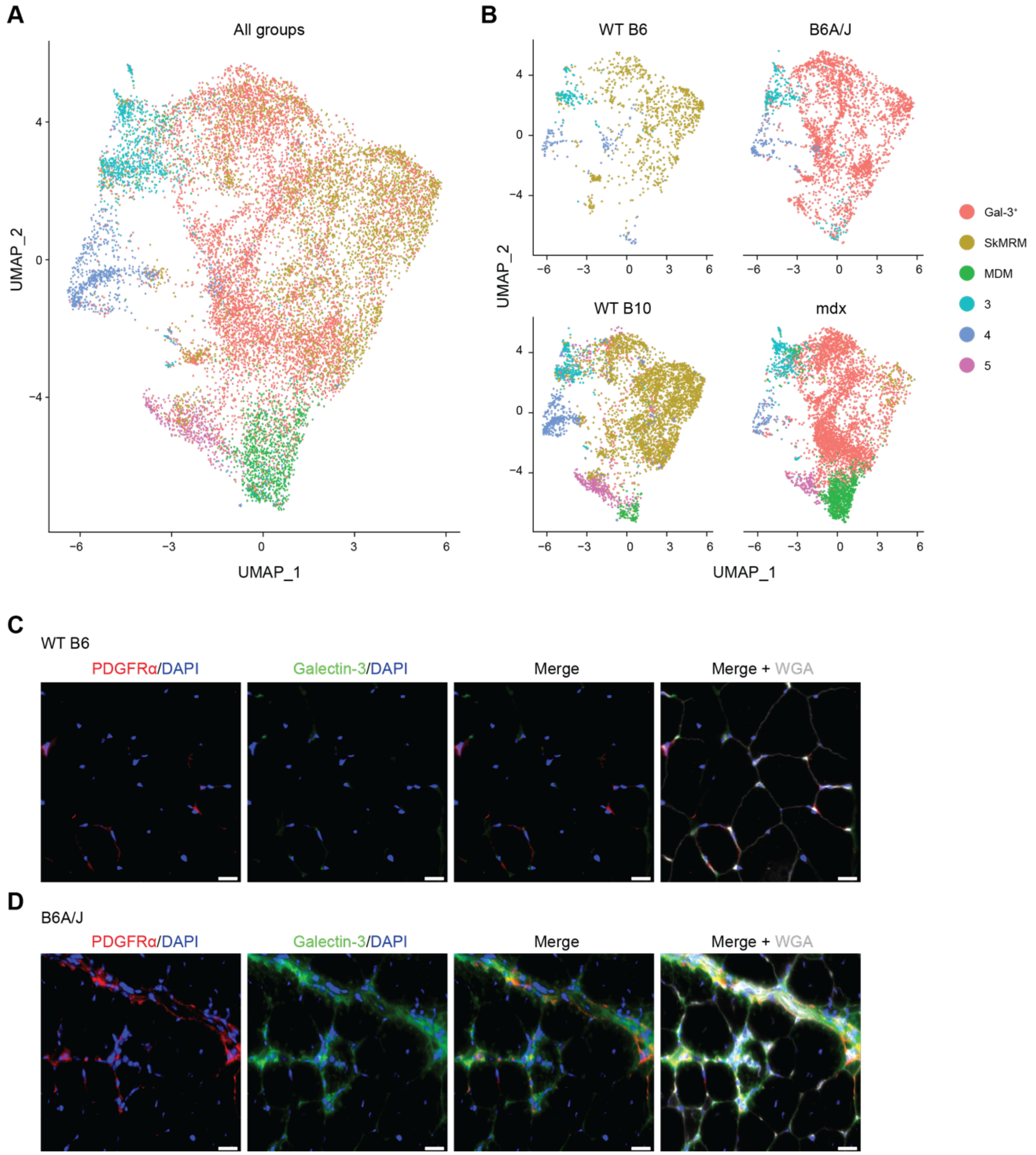

Fig. S10. Reference-based integration of B6A/J and mdx muscle macrophage scRNAseq datasets. (**A**) UMAP plot of combined muscle macrophages datasets from mdx (n= 1, 4-wk-old), B6A/J (n= 2, 8-mon-old, pooled), WT B10 (n= 1, 4-wk-old) and WT B6 (n= 1, 8-mon-old) control mice. (**B**) UMAP plots for each genotype showed a predominant muscle macrophage subset corresponding to Gal-3^{+} macrophages in B6A/J, while cluster 1 (SkMRM) is the dominant population in the matching B6 control mice. (**C** and **D**) Immunofluorescence staining of 12-month-old B6 (C) or B6A/J muscle (D) with PDGFRα (red) and galectin-3 antibodies (green). Frozen sections were counterstained with DAPI (blue) and wheat germ agglutinin (WGA) (white) to highlight nuclei and the ECM, respectively. Scale bar, 20 µm.

Supplementary Figure 11

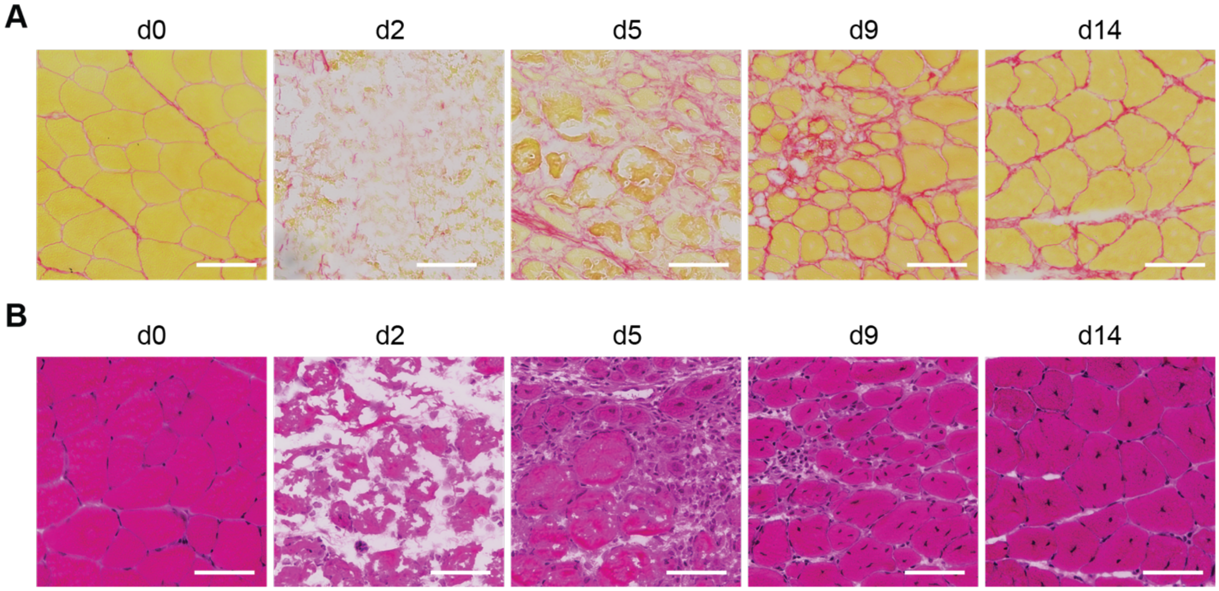

Fig. S11. Histological examination of acutely-injured muscle. (**A, B**) Muscle histology at different time points following $BaCl_2$-induced acute injury in 6-wk-old B6 mice. Representative picrosirius red (A) and hematoxylin and eosin (H&E) staining (B) of quadriceps cross sections. Scale bar, 100 µm.

Supplementary Figure 12

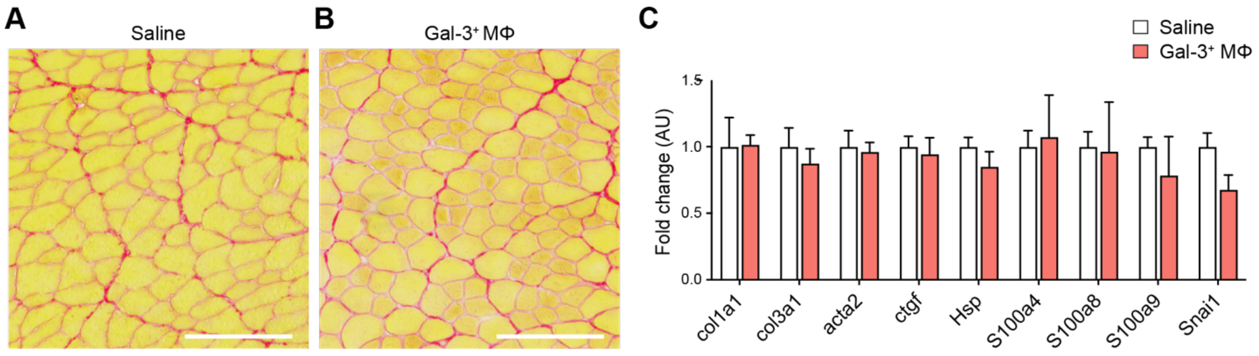

Fig. S12. Gal-3^{+} macrophages do not spontaneously promote fibrosis in healthy skeletal muscle. (**A and B**) Cross-sections of tibialis anterior muscles injected with saline (A) or gal-3^{+} macrophages (B) were stained with picrosirius red to interrogate collagen deposition. Scale bar, 100 μm (**C**) The expression of multiple genes associated with muscle fibrosis were measured by RT-qPCR in tibialis anterior muscles injected with saline or gal-3^{+} macrophages.

Supplementary Figure 13

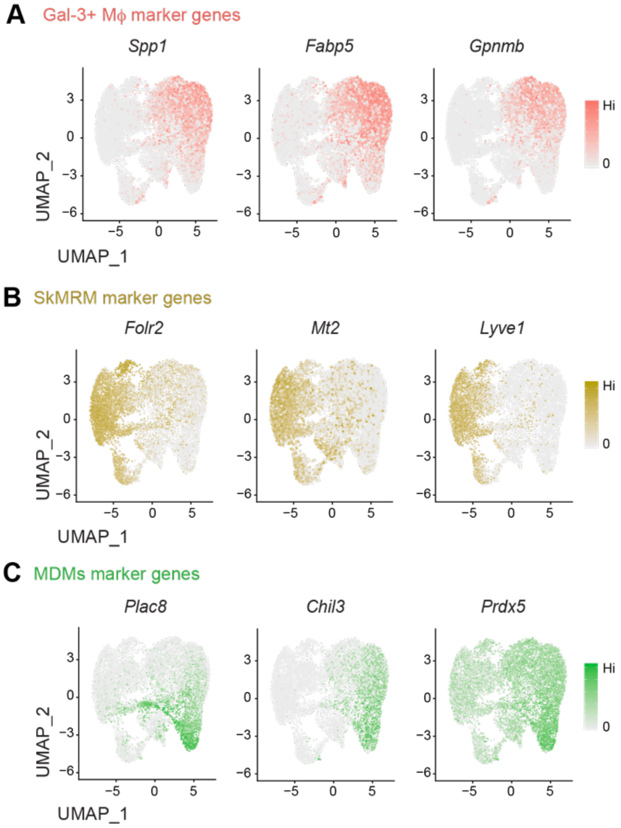

Fig. S13. Preferential expression of Gal-3$^+$ Mϕ, SkMRMs and MDMs marker genes. (A-C) Feature plots of macrophage marker genes in Gal-3$^+$ Mϕ, (A, Spp1, Fabp5, Gpnmb), SkMRM (B, Folr2, Mt2, Lyve1) and MDMs (C, Plac8, Chil3, Prdx5).

SUPPLEMENTARY TABLES

Table S1. Bulk RNASeq Summary Table (external table)

Table S2. Flow cytometry antibodies used in this study

Marker	*Color*	*Source*	*Clone*	*Dilution*
**Macrophage panel**				
Viability	Zombie-NIR	Biolegend	-	1:1000
CD11b	PerCP-Cy5.5	BioLegend	M1/70	1:100
F4/80	PE-Cy7	BioLegend	BM8	1:200
Siglec-F	BV421	BD Bioscience	E50-2440	1:150
Ly6c	BV605	BioLegend	HK1.4	1:300
CD52	PE	MBL	BTG-2G	1:3
Folr2	APC	BioLegend	10/FR2	1:200
Galectin-3	AF488	BioLegend	M3/38	1:200
**FAP panel**				
Viability	Zombie-NIR	Biolegend	-	1:1000
CD45	FITC	ThermoFisher	30-F11	1:500
CD31	FITC	ThermoFisher	390	1:300
Sca1	PE-Cy7	Biolegend	D7	1:5000
PDGFRα	BV605	Biolegend	APA5	1:200
Itgb3	PerCP-Cy5.5	Biolegend	2C9.G2 (HMB3-1)	1:100
Itgav	Biotin/SAV A647	Biolegend	RMV-7	1:200
CD44	PE	Biolegend	IM7	1:1000
